# Community recommendations on biobank governance: Results from a deliberative community engagement in California

**DOI:** 10.1371/journal.pone.0172582

**Published:** 2017-02-24

**Authors:** Sarah M. Dry, Sarah B. Garrett, Barbara A. Koenig, Arleen F. Brown, Michael M. Burgess, Jen R. Hult, Holly Longstaff, Elizabeth S. Wilcox, Sigrid Karina Madrigal Contreras, Arturo Martinez, Elizabeth A. Boyd, Daniel Dohan

**Affiliations:** 1 Department of Pathology and Laboratory Medicine, University of California Los Angeles, Los Angeles, California, United States of America; 2 Philip R. Lee Institute for Health Policy Studies, University of California San Francisco, San Francisco, California, United States of America; 3 Department of Social and Behavioral Sciences, Institute for Health and Aging, University of California San Francisco, San Francisco, California, United States of America; 4 Division of General Internal Medicine and Health Services Research, University of California Los Angeles, Los Angeles, California, United States of America; 5 Department of Biomedical Ethics, The University of British Columbia, Vancouver, B.C., Canada; 6 Genentech, Inc., South San Francisco, California, United States of America; 7 Office of Research Ethics, Simon Fraser University, Burnaby, B.C., Canada; 8 School of Population and Public Health, The University of British Columbia, Vancouver, B.C., Canada; 9 Independent Scholar, Torrance, California, United States of America; 10 Center for Clinical and Translational Science Institute, University of California Los Angeles, Los Angeles, California, United States of America; 11 University of California, Office of the President, Oakland, California, United States of America; University of Oxford, UNITED KINGDOM

## Abstract

United States-based biorepositories are on the cusp of substantial change in regulatory oversight at the same time that they are increasingly including samples and data from large populations, e.g. all patients in healthcare system. It is appropriate to engage stakeholders from these populations in new governance arrangements. We sought to describe community recommendations for biorepository governance and oversight using deliberative community engagement (DCE), a qualitative research method designed to elicit lay perspectives on complex technical issues. We asked for stakeholders to provide input on governance of large biorepositories at the University of California (UC), a public university. We defined state residents as stakeholders and recruited residents from two large metropolitan areas, Los Angeles (LA) and San Francisco (SF). In LA, we recruited English and Spanish speakers; in SF the DCE was conducted in English only. We recruited individuals who had completed the 2009 California Health Interview Survey and were willing to be re-contacted for future studies. Using stratified random sampling (by age, education, race/ethnicity), we contacted 162 potential deliberants of whom 53 agreed to participate and 51 completed the 4-day DCE in June (LA) and September-October (SF), 2013. Each DCE included discussion among deliberants facilitated by a trained staff and simultaneously-translated in LA. Deliberants also received a briefing book describing biorepository operations and regulation. During the final day of the DCE, deliberants voted on governance and oversight recommendations using an audience response system. This paper describes 23 recommendations (of 57 total) that address issues including: educating the public, sharing samples broadly, monitoring researcher behavior, using informative consent procedures, and involving community members in a transparent process of biobank governance. This project demonstrates the feasibility of obtaining meaningful input on biorepository governance from diverse lay stakeholders. Such input should be considered as research institutions respond to changes in biorepository regulation.

## Introduction

Academic medical centers, health systems, industry, and government are developing biorepositories (or biobanks) to collect and store samples and data from entire patient or community-based populations as a resource for advancing translational research, e.g., the national biorepositories established or in development in Iceland, the United Kingdom, and the United States (US)[1,2]. The scale and scope of these collections–as well as their inclusion of genetic information, linkage to electronic health records, and sharing across clinical, academic, and industry researchers–raises questions about how best to govern, or oversee, these research resources [1,3]. As the experience of Iceland’s Decode biobank illustrates, institutions that seek to develop patient- or population-based biorepositories must be responsive to patient and community perspectives on how to manage this research resource, with particular attention to ethical considerations related to consent, use of samples, and establishment of trusting governance relationships between researchers and subjects.

In the US, researchers, ethicists, and policymakers recognize that current standards for biobank oversight are often inadequate. Two high profile cases–that of Henrietta Lacks and the Havasupai tribe–have played a particularly notable role in illustrating the potential inadequacies of current regulatory and oversight frameworks [4,5]. The US Federal Government, via an Advance Notice of Proposed Rule Making (2011) and Notice of Proposed Rule Making (2015), is actively reconsidering how to improve its Common Rule protections of human research subjects [6,7]. Among other issues, they seek to align the Common Rule with current practices and future needs of biobank research by recognizing risks posed by advances in genomics, genotype-disease correlations that may require return of research results, unspecified future research, and large-scale health records research. The proposed changes to biobank informed consent practices also acknowledge current practice and emerging evidence that community members typically are willing to donate biosamples for research [8–10]. The notices reveal, however, that we have limited evidence on how to operate and oversee biobanks to assure that biobanks remain trustworthy and continue to enjoy patient and public participation [11,12].

To address this lack of evidence, we conducted a two-site deliberative community engagement to elicit recommendations from demographically- and linguistically-diverse Californians about how to govern biobanking at the University of California (UC). Deliberative community engagement (DCE) is a social scientific research method designed to elicit public responses to complex or technical issues. It has been used to examine community perspectives on biobank governance in the United States and globally, and it has also been used to examine other complex health issues such as the allocation of health care resources [13–19]. By accessing informed lay perspectives, DCE techniques effectively develop evidence to inform policy [15,20].

## Methods and design

This project was reviewed and approved by all appropriate Institutional Review Boards (IRBs); all participants read and signed an informed consent form before project activities commenced. The University of California, San Francisco, Human Research Protection Program Committee on Human Research approved this study, Engaging University of California Stakeholders for Biorepository Research (EngageUC) Consent Trial (Approval number IRB#14–13713).

We designed the community engagement as one component of EngageUC, a study funded by the US National Institutes of Health (NIH) that sought to identify opportunities and challenges in developing an ethical and efficient approach to biobanking within the UC system. In its mandate to explore ethical and efficient biobanking, EngageUC explored the possibility of developing a central UC Biobank, and it also sought to obtain recommendations on how to assure responsive governance of biobanking research throughout UC, whether or not a central UC Biobank was established.

EngageUC included five UC medical center campuses (UC Davis, UC Irvine, UC Los Angeles, UC San Diego, and UC San Francisco). The five campuses possess clinical data from approximately 13 million current and recent patients, largely drawn from California’s population of 38 million, which is notable for its diversity with respect to race and ethnicity, nativity and geographical ancestry, socioeconomic status and educational attainment, languages spoken and health literacy, and rural and urban residence [21]. While part of a single university system, the 5 UC centers are nevertheless geographically and administratively distinct and feature many distinct practices and policies related to research and biorepositories. A recent survey of the 5 campuses identified at least 440 active biobanks and 800 possible biobanks [22]. These UC biobanks have diverse aims, research interests, sample types, associated clinical data, sample sources and consent language depending on the specific type of research project.

A high priority goal for EngageUC was to obtain input from ordinary Californians about how to govern biobanking at UC in order to assure ongoing public trust and engagement in UC translational research. To achieve this goal, lay Californians would have to understand how biosamples and personal medical data could contribute to research findings, as well as the ethical concerns, regulatory issues, and existing institutional protections for participants. Additionally, given the diversity of biobanks across the five UC medical campuses, it would be necessary for lay Californians to consider different types of biosamples (e.g., blood, urine, remnant tissues), collected from individuals with varied states of health (e.g., healthy or with an acute or chronic illness) and collected at various points of contact with the health care system (e.g., during a routine medical office visit or during surgery).

Based on a review of established methods, we identified Deliberative Community Engagement (DCE) as a method likely to achieve the study goals: engaging lay participants on an unfamiliar and complex topic, eliciting their nuanced views and complex trade-offs, empowering them to provide decision makers with recommendations they developed, and minimizing the influence of outside “experts” on such recommendations. In a DCE, participants are considered the “experts” in how the topic at hand concerns or affects the population at risk—key information for decision-makers trying to balance social trade-offs in a technically complex undertaking. DCE methodology would allow lay Californians to understand how biosamples and personal medical data could contribute to research findings, as well as the ethical concerns, regulatory issues and existing institutional protections for participants. For example, given the diversity of UC biobanks, it was important for lay deliberants to consider different types of biosamples (for example, remnant tissues from surgical procedures, blood, urine, etc), collected from individuals with varied states of health (for example, healthy, chronic illnesses such as diabetes, or recently diagnosed life-threatening illnesses such as cancer) and samples collected a various points of contact with the health care system (for example, part of a routine medical office visit or during surgery). Other methods of engaging diverse participants, such as focus groups or Delphi methods, were judged to be less likely to achieve the overall study goals given the complex, technical issues involved in biobanking at UC.

The DCE method typically involves: recruiting a sample of relevant stakeholders to serve as deliberants; engaging in educational activities to ensure deliberants have a working knowledge of the technical issues at hand as well as social and other trade-offs; facilitating discussion so participants can clarify their own values and understand others’; developing and discussing specific recommendations related to issues important to the group; and, in some DCE, taking a final vote to provide an additional gauge of deliberants’ support for specific recommendations [14,23,24]. DCE has been used to examine a variety of health-related issues [14,20], and the DCE approach used in EngageUC has been published elsewhere [21]. This project was reviewed and approved by all appropriate Institutional Review Boards (IRBs); all participants read and signed an informed consent form before project activities commenced.

The residents of California constitute UC’s stakeholders, so EngageUC sought to assemble a representative group of residents to make recommendations about UC biobanking governance via DCE. Given the need for in-person deliberation, the sampling frame for the DCE included two large and diverse metropolitan areas (Los Angeles and San Francisco) and two language groups (English and Spanish), as well as variation based on race/ethnicity, age and educational background (below) in order to assure that diverse viewpoints on issues potentially related to biobanking were included. For the San Francisco event, the recruiting team made special efforts to include participants from less urban counties in the region. The design focused on community members, rather than specifically on patients, because biorepositories and biomedical research impact all Californians, because community members may at some point be UC patients, and because state residents are the ultimate stakeholder of the public university’s research resources including biobanks.

To complete the sampling frame, we invited participants from the 2009 California Health Interview Survey (CHIS) who had agreed to be contacted for future research studies to participate in the DCE. CHIS is a population-based health survey of approximately 48,000 Californian adults, teens and children carried out by the UCLA Center for Health Policy Research [25,26]. The largest state health survey in the United States, it is conducted continuously in two-year cycles in every county in the state and in five languages. Its publicly available data provides population-based estimates for the state, for counties with populations over 60,000 (41 of the 58), and for all major racial and ethnic group and some ethnic subgroups. It collects data on “essential health topics” every year (e.g., health conditions and behaviors, mental health, health care use) as well as emerging topics [26,27].

CHIS 2009 included 358 candidate participants (LA: 187; SF: 171) who met our study’s inclusion criteria. We recruited to fulfill our stratification quota: one to two people for each combination of the following categories: five racial/ethnic populations (African-American, Asian, Latino, Native American/Alaska Native, and White), three age groups (under 38, 39–56, over 75), and three educational backgrounds (less than high school degree, high school degree through bachelor’s degree, or more than bachelor’s degree). Once those quota were achieved, we continued to recruit participants using simple random sampling from the remaining CHIS participant pool until we had secured sufficient number of DCE participants. We contacted 162 of the candidates (LA: 84; SF: 78). Sixty-four of the candidates agreed to participate in the DCE study activities (LA: 33; SF: 31). Of these, 53 attended the first days of the events (LA: 27; SF: 26) and 51 (LA: 26; SF: 25) completed the DCE events (Table 1). Attrition among confirmed attendees (n = 2) was due to mental or physical illness.

**Table 1:** Participant Demographics Table.

	Los Angeles (n = 26)		San Francisco (n = 25)	
	Number	%	Number	%
Gender				
Male	14	54	10	40
Female	12	46	15	60
Language				
English	18	69	25	100
Spanish	8	31	0	0
Race*				
White	16	62	8	32
Black	4	15	5	20
Asian	1	4	7	28
Pacific Islander	0	0	2	8
Native American	4	15	2	8
Other or missing**	4	15	5	20
Ethnicity = Latino	10	39	6	24
Education				
<HS	6	23	0	0
HS	3	12	1	4
Some College	7	27	3	12
AA degree/vocational	1	4	4	16
College/BA	5	19	7	28
Master/PhD	4	15	10	40
	Mean (Std. dev.)	Range	Mean (Std. dev.)	Range
Age in years	49 (11.7)	31–70	50 (14.8)	23–73

* Participants are listed in each racial category that they reported. Therefore, the totals in this section of the table are greater than the sample size of each site and the percentages sum to greater than 100%.

** Participants’ reports of their “other” racial background included South Asian, Mexican, Belizean, Sud Americano, and Armenian. Additionally, one respondent in SF did not report information about race.

The makeup of the deliberant groups was similar but not identical to the demographics of the counties from which they were drawn (Los Angeles; San Francisco, Alameda, Contra Costa, San Mateo)[28]. LA deliberants had comparable levels of educational achievement to the larger population, but were more likely to be Black and Native American and less likely to be Asian or White. Compared to the demographics of surrounding counties, SF deliberants were more highly educated (86–92% vs. 100% with a high school degree, 39–54% vs. 68% with a Bachelors), more likely to be Black, Pacific Islander and Native American, and less likely to be Asian or White. Comparisons by race are imprecise, however, as EngageUC allowed individuals to report as many race categories as they wished.

We held DCE events in Los Angeles (LA) in June 2013 and in San Francisco (SF) in September/October 2013. Both consisted of full-day activities over two non-consecutive weekends (a total of 30 hours over four days). The LA event was bilingual in English and Spanish, and professional interpreters provided simultaneous translation for full-group discussions; one small group conducted discussions in Spanish and the remaining two small groups had English discussions. Participants received $100 per day for participation plus transportation costs. Per hour, this approximates the living wage required for an adult to support him- or herself in the San Francisco Bay area [29].

The study team provided briefing materials (hand-outs, a specially prepared briefing book, event website, and session handouts) in both languages as well as in audio format to participants two weeks before the event. The EngageUC study team drafted the briefing book based on previous experience conducting similar community engagement events [18], and worked with team members trained in community engagement to tailor it to ensure content was accessible and culturally appropriate for the diverse California communities of EngageUC. Content included discussion of issues that have concerned the general public or specific communities, such as the immortalized cell line produced from Henrietta Lacks’ cervical cancer cells and the research performed on blood samples that was not approved by Havasupai Tribe member donors (see S1 Appendix). Professional translators created a Spanish version of the book for Spanish-speaking participants, and the study team made audio recordings of the briefing books available on the website for participants with low literacy.

Events were guided by an agenda developed by the project team. The first day of both events was devoted to in-person presentations and discussions with biobanking experts on the topics raised in the Briefing Book (see S1 and S2 Tables for event agendas, speakers, and topics). The goal of the presentations and discussions was to expose deliberants to diverse viewpoints on topics related to biobank research and governance. During the middle two days of the event, deliberants spent time in both large- and small-group discussion settings. The general discussion topics were selected by the study investigators (e.g., “informed consent”), but the members of each groups were encouraged to discuss specific issues they felt were most important. A moderator trained and experienced in deliberative methods facilitated large group meetings and supervised three additional moderators who were trained to conduct small-group discussions. The small groups provided opportunities for all deliberants to engage each other in meaningful conversation, provided a forum in which deliberants could discuss and consider many different opinions, and allowed deliberants to explore whether their positions were rigid or if they would be willing to accept modifications suggested by others. To ensure that the small-group and large-group conversations reflected the viewpoints of deliberants–not experts–moderators were selected who did not have expertise in biobanking or biomedical sciences, and they were trained to not answer technical or topic-specific questions during the events. When participants had questions in small-group settings, they were encouraged to discuss with each other, to consult the briefing book, or to raise questions with investigators during the large-group discussions. All sessions were audio-recorded and experienced observers documented all discussions via detailed observational fieldnotes.

After each day of deliberation, the EngageUC study team, the large- and small-group facilitators, and the trained observers met to review and discuss topics that were discussed by the deliberants during the day. The goal of this review was to identify topics that engendered consensus or debate during the day. Drawing on fieldnotes and audio recordings, these topics were identified and summarized in form of recommendations to be voted upon, e.g. “UC biobanks should include oversight by a group of ordinary Californians.” To the extent possible, these recommendations included language used by deliberants themselves during the group discussions.

During the DCE final day large-group meeting, the lead facilitator reviewed all the potential recommendations that had been developed during the deliberation. Deliberants discussed and refined the language of each recommendation and then used an audience response system to anonymously vote on whether to endorse its final wording [30]. Following each vote, the facilitator invited deliberants to discuss the reasoning behind their vote. All propositions, discussions, votes, and justifications for votes were recorded for later analysis and became an important product of the DCE.

We conducted qualitative content analysis of the notes and transcripts from the final day of each DCE to identify all recommendations that were voted on by deliberants and to determine the final votes (Yes/No/Abstain) for each recommendation [31]. We used thematic analysis of the recommendation language and of the discussion of each vote to identify the substantive content of each voting recommendation. This analysis focused on the specific meaning that deliberants assigned to each recommendation (as indicated by the discussion of the voting language) as well as the reasons that deliberants voted for, against, or in abstention (as indicated by discussion of reasoning after the vote was taken). Based on this analysis, we ascertained which topics had been addressed at both DCE sites and which topics only arose at one site.

## Results

We identified a total of 57 unique recommendations from the DCE events (29 in LA, 28 in SF). 23 recommendations (10 in LA; 13 in SF; S3 Table) were similar and supported by the vast majority of participants at both LA and SF events (80% or more at each site). 30 recommendations were voted on at only one site, and 4 recommendations received high levels of support at only one site. Thematic analysis of the 23 highly-supported recommendations revealed 10 distinct themes in five topic areas. The topic areas, recommendations, voting patterns, and themes are shown Table 2 and discussed below.

**Table 2:** Analysis of highly supported cross-site recommendations from DCEs.

	Los Angeles		San Francisco		
Topic and Recommendation(s)^1^	Rec. ID#	Yes/No/ Abstain	Rec. ID#	Yes/No/ Abstain	Theme
**A. Public Education**			** **		
The public should be educated about bio-banking	LA3	25/1/0	SF3	25/0/0	The public should be educated about bio-banking
**B. Sample and Data Sharing**					
UC can share [should support sharing] samples [and data] among [all] researchers provided…					
…good oversight (SF)/ethical governance (LA)	LA6	25/0/1	SF7, SF8	22/2/1, 22/1/2	UC can share samples with other researchers provided that there is oversight and it is for the greater good.
…it advances research	—		SF7, SF8	22/2/1, 22/1/2	
…it is for the public good.	LA6	25/0/1	—		
If data/samples are shared (outside of UC [SF]), results should be shared back to…					Results from this sharing should inform future research.
… UC	—		SF9	24/0/1	
…Research programs and clinical organizations	LA7	23/2/1	—		
**C. Informed Consent**			** **		
Consent must [should] be obtained when donor is less [not] stressed, worried or preoccupied	LA9	24/1/1	SF12	24/0/1	The consent process should be initiated at a time of low stress for the patient by a knowledgeable, trustworthy individual; there should be ample time for discussion.
It should be obtained via interaction with a knowledgeable [and trusted] person [who has time to answer questions]	LA9	24/1/1	SF11	24/1/0	
Consent forms must [should] be written clearly and use simple language [and large fonts, in the donor's preferred language]	LA8	25/0/1	SF10	24/0/1	The format and language of consent materials should make the content easy to understand.
**D. Trustworthy Governance, Including the Community**				** **	
A community body must have a meaningful role in the oversight of [UC] biobanks.	LA1	26/0/0	SF2	24/0/1	The community should be represented by a body that has a meaningful role in the oversight of biobanks.
The community [including their needs, priorities] should be represented by a body.	LA2	25/1/0	SF1	22/0/3	
They should represent California's diversity	LA2	25/1/0	SF4	23/1/1	Members of this body should represent CA's diversity.
Oversight should be conducted by the community …	LA4	22/2/2	SF5	21/2/2	Oversight should be conducted by the community and other stakeholders.
…and by the IRB, the biobank, and UC.	—		SF5	21/2/2	
…and other unbiased stakeholders (e.g., scientists, medical professionals and lawyers)	LA4	22/2/2	—		
There should be monitoring and consequences for improper handling or misuse of data or samples.	LA5	26/0/0	SF6	23/1/1	There should be monitoring and consequences for improper handling or misuse of data or samples.
**E. Return of Research Results**			** **		
Individuals should be able to choose to receive results or not.	LA10	23/2/1	SF13	24/0/1	Donors should be able to choose to receive results or not.

^1^Brackets indicate content that appeared at only one site.

Note: LA and SF recommendations with similar content appear next to one another. In cases where recommendation content from one site overlaps with that of multiple recommendations from the other site (e.g., LA9), or features content that is not shared with the “matching” recommendation (e.g., LA6), the recommendation appears on multiple lines of the table.

### Topic A: Public education

Deliberants at both sites developed and voted on recommendations related to the need for public education. They nearly unanimously supported increased and improved public education about biobanks and their role in biomedical research (LA3, SF3)–a recommendation that arose at multiple points in the deliberation. Some deliberants commended the DCE process as an educational model, and many suggested public service advertising on television or radio. Deliberants endorsed the belief that education would help potential participants understand and accept biobank participation, potentially foster better-informed consent, and bolster trust in biomedical research at UC and beyond. The one deliberant who voted against public education was concerned about diverting funds from research activities.

### Topic B. Sample and data sharing

Deliberants endorsed sharing biosamples and data with researchers in academia and industry, as long as sharing was for the “greater good” (including for good research purposes) and was paired with strong ethical and regulatory oversight (LA6, SF7, SF8; see also LA5 and SF6, discussed below in “Trustworthy Governance”). In these recommendations, deliberants supported oversight bodies as the preferred entities to control which researchers and research projects accessed and analyzed samples and data. Consistent with this model, LA deliberants strongly supported the recommendation that “consent should be as broad as possible” (25/1), and SF deliberants expressed mixed support for the recommendation that donors “choose specifically how their samples will be used” (10/11/4). Deliberants who abstained or voted no on the sharing recommendations were concerned that such sharing could compromise UC’s ability to compete for funding or to make new discoveries. Some deliberants wanted different policies to apply to anonymous versus identified samples or for samples obtained with research consent versus samples obtained without such consent. Some felt that “good oversight” policies sufficed to guide sample sharing while others wanted to specify particular institutions or sets of institutions with which UC would be permitted to share. Additionally, deliberants felt that results from the sharing of data with other entities should be shared back with UC (SF9) and/or other research entities (LA7). Accompanying discussion highlighted the advantages of such “sharing back” to promote further research and discovery.

### Topic C. Informed consent

Both sites were nearly unanimous in recommending that consent forms be written in clear, simple language (LA8, SF10). Deliberants who abstained felt forms should include details in order not to misconstrue information or were skeptical that such a recommendation would actually succeed in changing consent forms. Most deliberants at both sites felt consent should be an interactive process carried out with a person knowledgeable about the proposed research (LA9, SF11). Some dissenting and abstaining voters wanted the recommendation to additionally stipulate that the potential donor must have full decision-making faculties and should not be under the influence of medication; others abstained because they did not want samples to be discarded solely because of who obtained consent, e.g. a physician versus a clinical research coordinator. Deliberants also endorsed that consent should be obtained at a time of low worry and stress (LA9, SF12). Persistent disagreement over this recommendation centered on concerns that obtaining consent at a time when a donor is not stressed may not be feasible. Some deliberants also wondered if consent obtained a long time before the sample was collected might mean patients would not be in the proper mindset to fully appreciate the risks and benefits of participation.

### Topic D. Trustworthy governance, including the community

Deliberants consistently supported the need for trustworthy and transparent biobank oversight or governance (these terms were used interchangeably) by multiple stakeholders (LA4, SF5). They envisioned community participation as an especially important component of biobank governance, endorsing several recommendations that mandated a meaningful role for a community body in the governance of biobanks (LA1, LA4, SF1, SF2, SF5). Deliberants at both sites supported the involvement of the community, scientists, medical professionals, lawyers, and UC itself in oversight (LA1, LA4, SF1, SF2, SF5). The handful of deliberants who voted against these recommendations were concerned that scientists’ oversight might be biased (though others noted that different viewpoints could be desirable) or were concerned that some groups (e.g., “UC”, “community”) were only vaguely defined in the recommendations. Some were unclear if a community body was needed in addition to the university’s IRB; some felt that UC researchers themselves could represent community views.

The vast majority of deliberants at both sites felt that people serving on such a body should be representative of the wider community’s diversity (LA2, SF4). Some participants, however, expressed concern that scientific research should not be overseen by lay individuals who do not understand the research, or worried that certain definitions of “community” could exclude too many stakeholders. Deliberants discussed ideas regarding how board members could be selected and rotated to ensure members fairly represented the underlying community, however, no specific community board nor specific governance mechanisms were defined.

Deliberants felt there should be monitoring and consequences for improper handling or use of biobank materials, including the abuse of resources or inappropriate release of information (LA5; SF6). In SF, some deliberants voted against this recommendation on the grounds that researchers who intentionally mishandled samples or data should receive harsher punishment than those who did so accidentally. Others did not support this recommendation because of its negative focus on punishment.

### Topic E. Return of research results

Deliberants at both sites recommended that patients be given a choice about receiving results from analysis of their samples (LA10, SF13). Deliberants felt that biosample donors had a right to know information that could improve their health or their family’s health or that might require some type of medical intervention. Those who disagreed noted that donated samples were for research not individual findings, or that returning results could be costly or operationally burdensome. Some deliberants also expressed concern that the personal implication of research results may be unknown or uncertain, particularly in the early stages of research, and that returning such results might be misleading or cause unnecessary concern for biosample donors. In the course of these discussions many deliberants supported the idea of the patient’s doctor or other medical professional overseeing and translating the return of results to the patient.

## Discussion

Using deliberative methods, EngageUC assembled a diverse, representative group of California residents to develop and provide recommendations on how to ensure ethical management of University of California biobanks. As in similar past engagements, deliberants demonstrated a capacity to understand and discuss biobank operations, articulate their own values, and develop, refine, and vote on dozens of specific recommendations [14,16,32]. EngageUC deliberants communicated support for the role and importance of biobanking at UC and developed and endorsed recommendations related to educating the public, sharing samples broadly, monitoring researcher behavior, using informative consent procedures, and involving community members in a transparent process of biobank governance.

These recommendations from the California DCE are notable for several reasons. First, the calls to educate the public about biobanking and biomedical research, and for the broad sharing of samples, are indicators of deliberants’ general support for biobanking at UC. The results suggest that an informed public may be interested in biorepository research and likely to support such research if it is conducted in a transparent and trustworthy manner.

Second, participants supported the use of oversight entities to provide ethical governance of the biobank and to ensure proper control and use of samples and data in research. EngageUC deliberants discussed but showed low support for other potential governance models such as *a priori* specification or restriction on sample or data use, or donor management of their own data and samples [33]. This is consistent with other findings of limited public support for granular individual self-management [34–38] and, importantly, it deviates from models of donor management that some biomedical research stakeholders have proposed [39,40]. Similarly, the DCE participants supported systems that would protect data and samples via monitoring and penalizing researchers—ideas that are gaining traction elsewhere as well [41]. Awareness of past ethical transgressions in genetic research [4,5] did not convince DCE participants to limit sample sharing or recommend a donor-managed model of access.

Third, our findings are consistent with those from previous DCEs that engaged more homogeneous and exclusively English-speaking deliberant groups, and with the Common Rule NPRM with respect to improving the consent process as well as underscoring the importance of transparent governance that includes community representation [7]. This consistency may indicate genuine public support for such steps.

To translate these recommendations into a biobank governance plan requires soliciting input from additional stakeholders and biobank leaders. It also is important to note the limitations of the current study. Some limitations were unavoidable in order to successfully employ the DCE method. We engaged a small number of participants, and we included only English- or Spanish-speaking deliberants from communities in California’s largest metropolitan regions. SF-based participants were more highly educated than the region’s population, and all DCE participants had previously agreed to participate in health-related research. Though we excluded individuals who had extensive knowledge of biobanking, our participants may have been more interested in health- and research-related matters than the average Californian. Additionally, the biorepository-focused expertise that deliberants gained immediately before and during the DCE distinguished them from the average Californian. However, while these limitations had the potential to generate a bias towards endorsing UC biobanking, it is worth noting that deliberation at both DCE events included substantial amounts of critical discussion of biobank research and practice. This included concerns about profit-driven research and regarding conflicts between donor protection and scientific expedience. The study strengths include engaging a multi-lingual and racially-, ethnically-, and socioeconomically-diverse set of participants; similar DCE conducted previously had not been as inclusive.

Finally, this paper includes only highly-supported recommendations. Policymakers may also want to consider recommendations that were not highly supported. DCE discussions of these more contested recommendations were often extensive and highly complex; there is not adequate space for their analysis here.

## Conclusion

Researchers are recruiting larger and more diverse populations to advance contemporary biomedical research for “big data” studies in precision medicine, genetics, and comparative effectiveness. The governance of samples and data in these large repositories introduces new challenges, and in response regulators have proposed far-reaching legal changes. In an era where healthcare data breaches are common and surveillance by government and commercial entities appears unavoidable, the biomedical research community may be well-served to proactively build and maintain a trusting relationship with the public, who constitute its research subjects and beneficiaries, above and beyond what is required by lawmakers. One way to do this is to directly gauge community views of biobank governance in addition to seeking expert opinions of scientists, clinicians, IRBs, and ethicists.

The strongly-supported recommendations from this DCE suggest the participants value a relationship with the biomedical research community that goes beyond merely providing consent to participate in studies. Given the study design it is possible that the wider public in California also shares this attitude, though more research is needed to confirm it. Though these recommendations are neither radical nor unprecedented, it is noteworthy that NIH, the President, other US Federal agencies, the State of California and academic institutions within California have not funded or mandated programs that would satisfy them. As they represent diverse communities in California and echo outcomes from deliberations elsewhere, we propose that the EngageUC recommendations should be considered actionable evidence to guide development of modern biorepositories as an ethical and sustainable research resource.

## Supporting information

S1 Appendix Briefing Book. (PDF)

S1 Table Agenda for Los Angeles Deliberative Community Event. (PDF)

S2 Table Agenda for San Francisco Deliberative Community Event. (PDF)

S3 Table Deliberant-Generated Recommendations & Vote Counts. (PDF)
