# Supplementary material for: Community recommendations on biobank governance: Results from a deliberative community engagement in California
Source: PLoS One. 2017 Feb 24;12(2):e0172582. doi: 10.1371/journal.pone.0172582 (PMC5325297; doi:10.1371/journal.pone.0172582)
Supplement: S1 Appendix — (PDF) [file pone.0172582.s001.pdf]

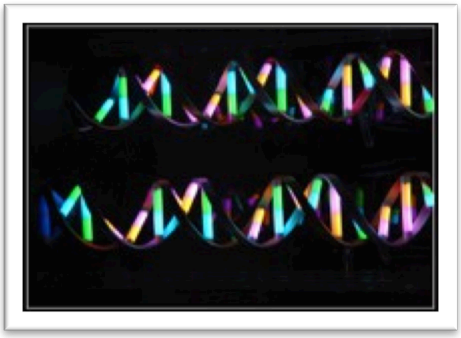 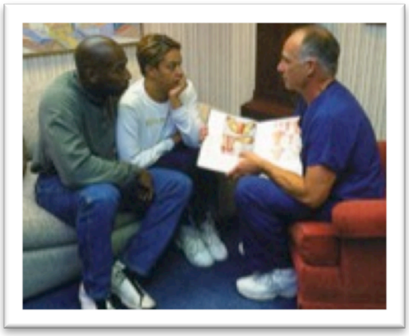 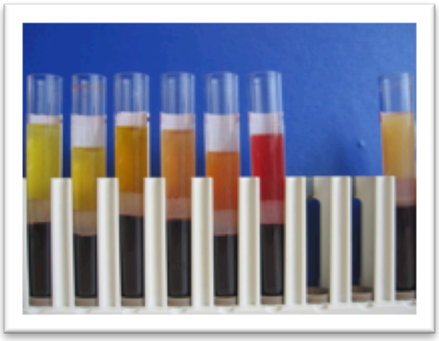

Biobanking at the University of California

A deliberative community engagement

An **EngageUC** Project

EngageUC is a collaboration that brings together researchers, health care providers, University of California leaders, and community members

Supported by the National Center for Advancing Translational Sciences (NCATS)
at the National Institutes of Health (NIH)

NATIONAL INSTITUTES OF HEALTH

NIH National Center for Advancing Translational Sciences

Purpose of this Book

The purpose of this book is to help you think and talk about biobanking. This information will help prepare you to participate fully in the Deliberative Community Engagement. **Please read the book before you arrive on the first Saturday.** This book explains what a biobank is, how biobanks work, and what kinds of research use biobanks.

In the following pages, we:

- review how samples of blood and tissue are collected and linked to health information,
- explain the process of asking patients' permission to use their samples,
- consider the potential benefits of research using biobanks,
- describe how biobank samples and health information are protected, and,
- discuss the impact of biobanking on California communities.

We have done our best to include a broad range of perspectives on biobanking. However, we realize that this book cannot include all opinions on the topic. Rather than trying to provide the "right" answer, the aim is to encourage you to find your own view and raise other issues that are important to you.

The results of the Deliberative Community Engagement will be available online for members of the California public to review. Other members of the community will be able to provide input.

A glossary (definitions) of biobanking terms is included at the end of this booklet. For example: "A biobank is a collection of biological samples of human blood and tissue for use in research. Samples are frozen or stored to be used at a later time. Biobanks are critical resources for research."

A Deliberative Community Engagement

EngageUC is a collaboration that brings together researchers, health care providers, University of California leaders, and community members to:

- create community-guided governance for biobanks across the 5 University of California medical campuses (Davis, Irvine, Los Angeles, San Diego, San Francisco);
- develop policies about the management of leftover blood and tissue samples collected for research; and
- redesign the process of asking patient permission for research use of leftover biological samples, including research using genetic information.

EngageUC will inform biobanking policy. The project includes several research studies, all focused on building better biobanks in California. For example, the EngageUC team will be conducting a study of how best to ask patients' permission for biobank research.

In 2013, EngageUC will hold two Deliberative Community Engagement events—one in Los Angeles and one in San Francisco —bringing together Californians from diverse backgrounds to offer advice to the University of California about research biobanks. At these events, participants will hear from scientists, doctors, patient advocates, and research leaders. They will discuss research using human tissue and blood samples that may be linked to a person's health record or information.

The topics will include:

- What is biobanking and how does it work?
- What are the benefits of research using tissue and blood samples?
- What protections are in place for the privacy and security of health information and samples?
- Why are some people concerned about sharing samples with researchers?
- How do research institutions in the U.S. inform people about tissue donation? How do they ask permission to use samples and health information?
- How does the University of California oversee biobank operations?
- What role should community members play in the oversight of biobanks?

Invited members of the general public will talk together about biobanking research, consider all the options, and then make recommendations to the University of California. The EngageUC team will also be studying and evaluating the effectiveness of the community engagement events.

More information is available at www.EngageUC.org

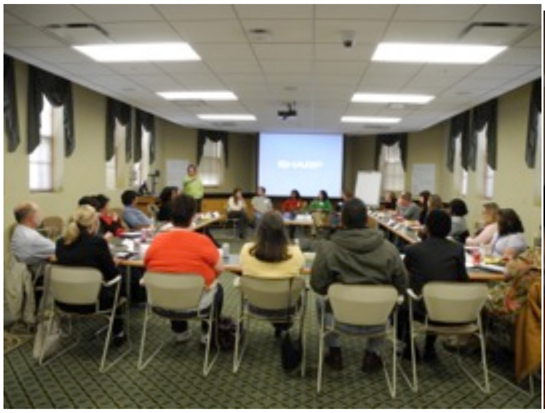

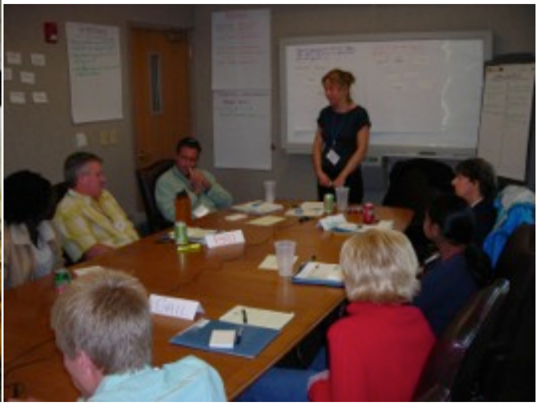

Table of Contents

1. What is “Deliberative Community Engagement?” 1

2. What is Biobanking? 2

3. The Potential Benefits of Biobank Research 5

4. Debating Biobanks 7

5. Oversight of Biobanks 9

6. Informed Consent 11

7. How are Privacy and Confidentiality Protected? 12

8. What Privacy Rules Apply to Biobank Research? 14

9. Should Results Discovered in Biobank Research be Offered to Participants? 16

10. Biobanks and California Communities 18

11. Commercialization, Benefit Sharing, and Ownership 21

12. What Role Have Communities Played in Biobank Oversight? 22

13. Future Governance of University of California Biobanks 23

14. Glossary 25

15. Acknowledgements 28

1. What is "Deliberative Community Engagement?"

People in California are concerned about the conduct and regulation of biomedical research and technology. However, discussion about these concerns is shifting. In the past, experts often told people what they needed to know about research and technology. Today, we recognize that all people should have a voice in decisions about how research is conducted. In particular, Californians should have a say in policies about the use of biological samples and health records for research.

In 2013, EngageUC will hold two Deliberative Community Engagement events: one in Southern California and one in Northern California. The goal is to bring together Californians from diverse backgrounds to offer advice to the University of California about research biobanks. We call it a "deliberative" community engagement because our event is based on principles of "deliberative democracy." That means that we seek to increase participation of Californians and create a place where people can talk and debate together.

Purpose of Community Engagement

In these community engagement events we aim to do two things: educate and seek advice. Our goal is to improve research policies by drawing on people from many different backgrounds who have many different opinions and needs. By using the knowledge, insight, and advice of California residents, we can make better decisions and make sure that our research deserves public trust.

Community engagement events allow members of the general public to come together to talk about an important issue, consider all the options, and then make recommendations. "Deliberants" are people who participate in the community engagement. Deliberants are selected at random from the California population.

What Will Happen During the Community Engagement?

Deliberants spend two full weekends together in discussions about how blood samples and leftover tissue samples should be used and stored for research. This includes samples that remain after surgery.

On the first day of the event, there will be a series of educational discussions. Participants will learn about biobanks and will be able to ask biobank researchers questions. On the other days, sometimes all deliberants will meet together, and sometimes they will meet in smaller groups. All groups will be led by professional leaders. By the end of the second weekend, the full group will make recommendations about policies for research biobanks at the University of California. The events will include trained interpreters so that Californians who prefer to speak Spanish can participate fully.

The purpose of the EngageUC events is to help leaders at the University of California develop the best possible policies about research with human samples and data.

Past Community Events

Deliberative community engagement has been used before to help develop biobank policy. Residents of Minnesota advised the Mayo Clinic. People from the Canadian province of British Columbia and citizens of Australia gave advice about government-sponsored biobanks. Links to information about those biobank projects can be found on the EngageUC website.

Please Discuss the Issues at Home!

Our hope is that you will read this book and discuss it with your family and friends. Start a conversation about how you and your loved ones would want to be informed about the use of your blood and tissues in research. We look forward to exploring options for how samples from human tissue and blood should be shared and how patients will be informed about the use of samples.

The Deliberative Community Engagement Events are Part of the EngageUC Project

The EngageUC Deliberative Community Engagement events are part of a larger program that aims to improve how scientists at the University of California manage biobank samples and research. At the same time that members of the community are talking together about biobanking, groups of scientists, clinicians and University of California leaders are working together to develop biobank policy and governance. Learn more about EngageUC (Engaging University of California Stakeholders for Biorepository Research) by visiting: **www.EngageUC.org**. An audio recording of this book can also be found there.

2. What is Biobanking?

A biobank is a collection of biological samples of any type of human fluid (such as blood or urine) or tissue (such as a piece of tumor) for use in research. Samples are frozen or preserved and stored so that they may be used at a later time for research. Biobanks can include samples from people with particular conditions, like cancer or Alzheimer's disease, or biobanks might store samples from healthy people. Biological samples are often linked to health information from the medical record. The health information makes the samples more valuable to researchers. It allows researchers to make comparisons over time, for example studying how a disease progresses or whether a treatment works. Biobanks are used for many different kinds of health research, including genetic research. Examples of the health benefits of biobank research are provided later in Section 3.

How are Samples for a Biobank Obtained?

Biobanks are created in a number of ways. Some biobanks are created by doctors who study a particular disease. They may create biobanks by asking their patients for permission to collect and store samples and data for research. Sometimes, but not always, people volunteer to be in biobanks. The rules about when specific permission

must be obtained are explained later in Section 8.

Some biobanks are created with "leftover" blood or tissue. Many patients will have tests performed on their blood or other bodily fluid (such as urine) as part of their routine care. Often, some fluid remains after all testing has been completed. Similarly, when patients have tissues removed during diagnosis (a biopsy) or for surgery, some tissue may be leftover after testing is completed. These leftover body fluid and tissue samples are typically thrown away by the hospital, following laws and regulations for their safe disposal, but they may be preserved and stored in a biobank. When leftover tissues or blood are stored in biobanks, patients do not have to have another procedure, like a blood draw. They do not need to return to the hospital or clinic. This makes it easier to participate in research. However, not all patients may want to have their leftover tissue or blood used in research.

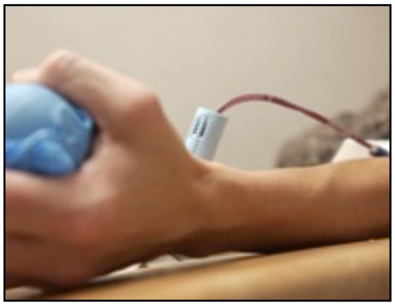

For many years, doctors and researchers saved leftover tissues in biobanks without asking specific permission from patients. Laws required that samples in these biobanks be stored in one of two ways: 1) Samples were anonymized, meaning that no one knew the identity of the patient who contributed the tissue. No links to patient data were kept. 2) Samples were deidentified, meaning that the patient's name was replaced by a code number, but a link was kept with the patient's name. We explain deidentification in more detail in Section 7. Today, thousands of tissue samples remain in biobanks.

Some biobanks include only samples. However, leftover fluids and tissues are more valuable when researchers know medical information about the patient who provided the sample. For example, asthma researchers might be interested in studying differences in blood levels of known pollutants in patients living close to highways and comparing those to patients living away from highways. Or, a researcher may be interested in testing colon cancer tissue to see if patients with one form of cancer live longer. In these examples, researchers would need access to detailed health information as well as blood and tissue samples.

Some biobanks are created when healthy people are asked to be part of a large collection of samples and data. They give a blood sample specifically for the biobank. Some biobanks are created to represent a particular population. For example, the United Kingdom has a large biobank with 500,000 samples. Another way of creating biobanks is to invite people who have certain diseases. For example all patients with breast cancer might be invited to be in a biobank to study that disease. In these types of biobanks people are generally asked to give specific permission to include their samples.

There is one additional kind of biobank: an "opt out" biobank. In opt-out biobanks, an institution may decide that everyone should be in the biobank unless they specifically say no. This is different than biobanks that require a formal process of asking patients' permission before storing samples. The biobank at Vanderbilt University provides detailed community education to

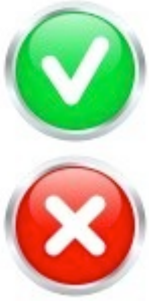

its local population and then allows people the opportunity to say "no" or "opt out" of biobank participation. This generally happens when they first become a patient. They are given the opportunity to say yes or no, but "yes" is the default position. That means patients are in the biobank unless they specifically say "no."

Why Are So Many Specimens Needed?

There are many reasons that researchers need large biobanks. Often, large quantities of data and samples must be studied in order to determine the actual cause of a disease. Including only a limited number of volunteers in a biobank gives an inaccurate picture of how common a disease is. Large biobanks are also needed to study rare diseases, or rare but deadly side effects of a common drug.

Today's technologies require "big data." Ten years ago the human genome was fully mapped for the first time, creating a unique research tool. Today, scientists use that map to study the link between genes and diseases by looking at a person's complete DNA sequence. Researchers study how very small genetic differences lead to cancer, heart disease, or other illnesses. To find these small differences, they need samples from tens of thousands of people. Biobanking allows for the collection of samples from many people and makes such research possible.

Does the University of California (UC) have Biobanks?

Currently, many biobanks exist at UC hospitals and clinics throughout the state. Some biobanks are specific to diseases like cancer; others are used for a range of studies. These collections are critical resources for UC research. Today, biobanking policies and procedures can be quite different from campus to campus, and even from lab to lab on a campus. The University of California hopes to develop a system that creates uniform policies to better protect patients. It also hopes to promote sharing of resources from biobanks because research today requires large numbers of samples.

Today's medical knowledge exists because of past research. Because the University of California is a public university system, supported through the tax dollars of the citizens of California, it is very important that UC keep the public's trust. UC has an obligation to use the public's money in a responsible, ethical way. Without the community's trust in researchers and institutions like UC, medical research cannot move forward. That's why getting community input on how to run biobanks, especially how best to look after people's samples and information, is so important.

Governments around the world are creating biobanks, as are hospitals, health systems, and private companies. National biobanks, such as the United Kingdom Biobank and the Estonian Genome Project, involve samples from hundreds of thousands of people. Hospitals all over the country have biobanks in the form of blood and tissue collections. In California, your trip to the hospital for surgery might end with your tissue being stored in that hospital's biobank.

The table on the next page presents examples of biobanks around the world.

Examples of Biobanks

Project	Location	Description
Marshfield Clinic Personalized Medicine Project	Marshfield, Wisconsin	One of the largest population-based genetic research resources in the U.S., involving over 20,000 central Wisconsin residents.
Genetic Alliance Biobank	Washington, D.C.	Created by patient advocacy groups to promote research. Recruits donors to the bank from the advocacy organization's community.
Icelandic Biobank	Reykjavik, Iceland	Blood samples from 270,000 Icelandic citizens and linked to Iceland Health Sector database and genealogical database
UK Biobank	UK	DNA, medical records and lifestyle questionnaires from 500,000 adult volunteers, to be followed for 30 years
Biobank Japan	Kanagawa, Japan	DNA samples from 300,000 individuals of 20+ years of age suffering from 30 common illnesses
LatinBanks	Various sites in Latin America	A partnership of link and regulate the biobanks in Latin American countries, such as Mexico, Costa Rica and Argentina

3. The Potential Benefits of Biobank Research

Biobank research has many potential benefits. Research teams have already had successes using human samples and health information as a resource. Some researchers work to help us understand particular diseases. Others are helping to shape "personalized" or "precision" medicine.

Biobanks and Cancer Research

The National Cancer Institute (a branch of the National Institutes of Health) funds biobanks devoted to many different cancers. In fact, "Comprehensive Cancer Centers" are required to maintain these resources. For example, the University of California, Los Angeles hosts projects focused on prostate, melanoma, and brain cancer, and the University of California, San Francisco hosts biobanks focused on breast, pancreas, prostate, and brain cancer. Cancer biobanks allow researchers to study why people develop the disease. Biobanks also help doctors develop new treatments and study how patients respond to treatment.

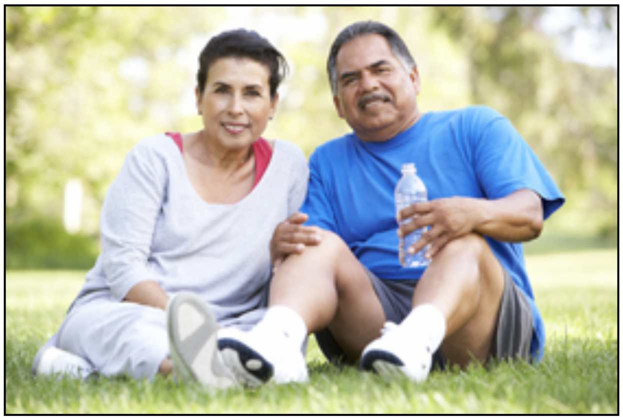

Biobanks Speed up Research

Biobanks are a convenient shortcut to medical discovery. They allow scientists to conduct health research without having to seek out and find new participants for each new project. They are like a library that puts together many books in one place. Research with these collections could:

- refine what we know about disease,
- discover environmental hazards,
- invent new diagnostic tests, and,
- determine why people respond differently to drugs.

Changing Our Understanding of Brain Tumors

Human samples linked to health information have been useful in helping to understand causes of brain tumors. A team at UCSF recently discovered new genetic links to a type of deadly brain cancer called glioma. There is no cure for glioma, treatments are complex and invasive, and no one knows how to prevent the tumors from developing.

In 2009, a team led by two UCSF researchers discovered new genetic markers that raise a person's risk of developing glioma. Some of the markers increase a person's risk of getting a specific kind of glioma. Other markers increase risk for all types of glioma. The study used genetic information from the stored blood of people with and without a history of brain tumors. It also used tumor samples that had been collected and stored since 1991 when one of the researchers began the study. Understanding these markers may help scientists understand how gliomas develop. In the future, the research may also help doctors develop new treatments.

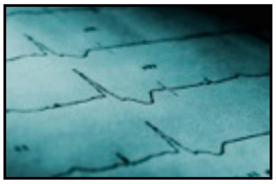

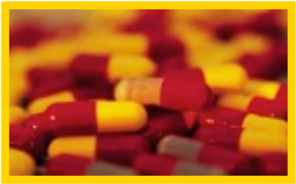

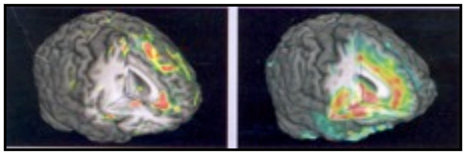

Creating Effective Treatments for Breast Cancer

A UCLA research team developed an important treatment for breast cancer using breast cancer tumor samples and data from medical records. The team took samples from patients' breast tissue and looked at the genes that control how cells grow. When they compared patients whose tumors had changes in one particular gene with other patients, they noticed differences. Patients with mutations in the Her-2 gene had worse outcomes. Their cancers came back sooner and spread more quickly. Treatments like chemotherapy and radiation did not work as well in these patients. They died sooner. This was an important finding, since mutations in this gene are seen in about 30% of all breast cancer tumors.

Over the next 12 years, scientists worked to develop an antibody called Herceptin. Patients with Her-2 mutated tumors who are treated early with Herceptin show much better survival than those not treated with Herceptin. Because it is an antibody, Herceptin is much less toxic than older forms of chemotherapy for breast cancer.

Determining How Different People Process Drugs

Pharmacogenomics is the study of how drugs work in the body. Biobanks are key resources for learning how genes and drugs interact. We know that some people are short and others are tall. Some have curly hair, others have straight hair. In the same way, people respond to medication in different ways. Drugs work better or worse, or may cause severe side effects in some people, while others are fine. Our genes partly determine height, eye color, and other characteristics. In the same way, genes affect whether our bodies process drugs quickly or slowly.

The drug "Warfarin" is a good example. Warfarin, also called *Coumadin*, is a

commonly used blood thinner, prescribed to keep blood clots from forming. In some people, the drug is quickly processed and eliminated from the body. In other people the drug is processed slowly, building to dangerous levels. In those people, the blood becomes dangerously thin and life-threatening strokes can occur.

Researchers use biobanks that include genetic information linked to medical records to study serious drug reactions. This has helped researchers find the actual genes that determine how drugs are processed in the body. Doctors are now able to use this information to make sure drugs are prescribed in the correct dose. This makes medications safer.

National Health and Nutrition Examination Survey (NHANES)

Biobanks may also support public health. An example is NHANES, a yearly survey conducted by the National Center for Health Statistics at the Centers for Disease Control. The survey combines biological samples, interviews and physical examinations to assess the health and nutritional status of adults and children in the U.S. Survey results are used to see how widespread major diseases are and who might be at risk for developing a disease. NHANES findings are the basis for developing public health policy and strategies for improving health nationwide. For example, outbreaks of flu can be tracked, and public health officials can figure out which groups in the population have already developed immunity to a certain strain of flu virus.

4. Debating Biobanks

Biobanks are critical for research, and people have been debating the ethics of biobanks for many years. If you ask scientists, lawyers, ethicists, policy makers, patient support groups and industry, many see great hope; others see cause for concern. Debates in the media focus on privacy. People have raised concerns about how samples and data are collected, stored, and shared.

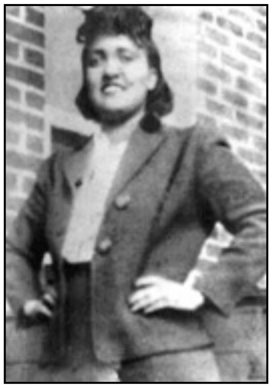

The Case of Henrietta Lacks

Recently, one case has received a great deal of attention in the media. It highlights many of the concerns raised about the use of human tissues. Henrietta Lacks was an African American tobacco farmer from southern Virginia who had cervical cancer at age 30. In the 1950s, one of Henrietta Lack's physicians at Johns Hopkins University Hospital removed a piece of her tumor and, without telling her, gave it to a group of scientists trying to figure out how to get human cells to continue growing once out of the body. At that time, obtaining permission before taking samples was not common practice.

Henrietta Lacks' cells became an extremely important research tool because they lived indefinitely in culture dishes in the lab. Named after her and nicknamed "HeLa," Ms. Lacks' cells were used in many kinds of medical research, including developing the polio vaccine. Neither she nor her family was ever notified of the HeLa commercial cell line or their contribution to these medical discoveries until 20 years after Ms. Lacks had died.

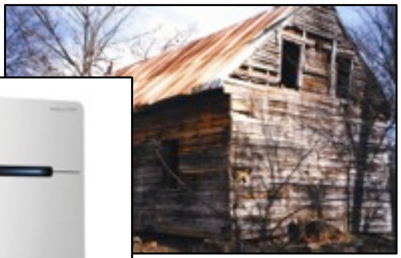

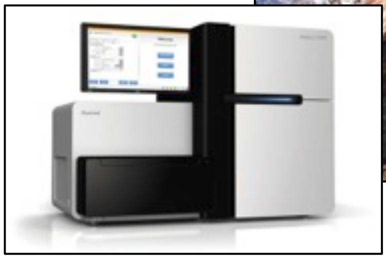

The story of Henrietta Lacks, her family, and her cells, is the topic of a 2010 best-selling book by Rebecca Skloot, *The Immortal Life of Henrietta Lacks*. Her story has focused concern on how scientists manage human tissue collection. It suggests the importance of respect for patients, especially those who are disadvantaged. It raises questions about how patients should be asked permission to use their tissue. It also raises the question of whether patients or their families should benefit when discoveries that lead to commercial products, such as HeLa cells, are made with their samples.

The story of HeLa cells does not stop there. HeLa cells are widely available; they are a basic research tool. In 2013, a group of European scientists used Henrietta Lack's cells to create a full copy of every piece of her DNA. This is called whole genome sequencing. It has only recently become possible to do this. Her genetic information was posted on the Internet, but later was taken down. Because her cells were widely available, the scientists who published Henrietta Lacks' genetic information did not get permission from Ms. Lacks' family to study her cells. Some scientists voiced their concern, as personal medical information about the Lacks family might be revealed through whole genome sequencing. They worry that this would also damage public trust in science. This example shows how regulations about privacy must be reevaluated in an era of genetic research.

Can We Learn from the Story of Henrietta Lacks? What Concerns do Biobanks Raise?

Henrietta Lack's story reveals the main dilemma of biobanking. Biological samples can lead to important discoveries, like the polio vaccine. But careful attention must be paid to the interests of donors. And potential cures must be balanced with respect for patients and their families. This story raises concerns about:

- **Privacy and confidentiality**. Henrietta Lacks had a type of cancer that conveys stigma. When they have a stigmatizing condition or disability, people fear harm if the information is revealed. Henrietta Lack's private health information was not protected by the doctors taking care of her.

- **Financial benefits**. The Lacks story raises questions of social justice. Who will benefit from research discoveries? Some people are critical of pharmaceutical companies who might use biobanks to develop new products and make profits. Others believe the companies are important to help research move forward and are enthusiastic about opening up biobanks to private industry.

- **Vulnerable populations**. Henrietta Lacks was the descendent of slaves and came from a background of extreme poverty. Her story illustrates that some people are vulnerable and may need special consideration as research policy is developed.

- **Permission to use samples**. No one asked Henrietta Lacks for permission to use her samples in research. Obtaining informed consent for research was not done when Mrs. Lacks was treated. Use of leftover tissue samples for research was common. A central issue in biobanking policy is how to obtain the permission of the individuals whose sample is being collected. This is often referred to as the "informed consent" process. However, since it is not possible to predict all the ways biobank samples might be used in the future, it is not possible to tell people exactly how their tissue will be used. Re-contacting donors for each new study is difficult and costly. Asking people for their permission for any possible future use of a sample raises a concern about whether informed consent is real.

- **Data Sharing and Return of Results**. Samples were shared widely with other researchers and new genetic technologies were applied to Henrietta Lack's tissue sample many years later. The story reveals how new technologies and research standards challenge biobank policy. People can be identified by their DNA. Also, unexpected findings raise concerns about whether and when results should be returned to participants.

The University of California is working to establish specific policies to address the types of concerns raised by the Henrietta Lacks story. To design good policy, scientists, clinicians, and university leaders need to better understand the views and concerns of the public.

The next sections of the booklet describe how biobanking research is currently overseen in the U.S. It reviews regulations for research with human subjects and laws protecting privacy, discusses concerns about California communities, and intellectual property. As you read these sections, ask yourself: Are current protections adequate? What role should the community play?

5. Oversight of Biobanks

Federal Human Subjects Protection Regulations

In the U.S., research that involves people (human research "subjects" or "participants") must follow certain regulations made by the federal government. The rules were designed to protect the participant's right to know what a research study is about, to make sure his or her participation is voluntary, to know what risks and benefits are involved, and to be treated fairly.

Research using biobanks is governed by these same ethical principles, which are sometimes referred to as "The Common Rule." Many branches of the U.S. federal government observe the same "common" rules to protect people in research. These regulations are like laws – all researchers must follow them. Research studies are reviewed once a year to be sure that the rules have been followed. The goal of the regulations is to reduce the chance that a research participant will suffer harm. If a serious problem is discovered, a research project is shut down or procedures are changed.

Role of the Institutional Review Board (IRB)

At universities like UC, it is the responsibility of a special committee, called an Institutional Review Board (IRB), to review and oversee the conduct of all research with human participants. IRBs are independent committees who are charged with protecting research participants' interests. The IRB is one of the key ways that UC oversees biobank research.

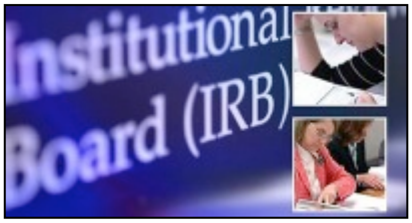

Independent IRB's help assure public trust in medical research.

An IRB is made up of doctors, nurses, pharmacists, scientists, ethicists, and people from the local community. Most studies that involve people must first be reviewed and approved by the IRB before starting research. The committee reviews studies to ensure that they are well-planned and ethical and to assure that appropriate steps are taken to protect the rights and welfare of people participating in research. IRBs also consider any special concerns of participants, such as Native Americans or vulnerable groups such as prisoners.

IRBs consider the following:

- look at how research studies are designed, how participants will be asked to join,
- how the risks and potential benefits of the research are described,
- how participants' privacy and confidentiality will be protected, and,
- whether procedures are in place for subjects to give informed consent.

In summary, the IRB is responsible for making sure that all rules and laws are followed. (The informed consent process is discussed below in detail.)

IRBs and Biobanks

Before beginning a biobank project a researcher writes a proposal and submits it to the IRB. IRBs are responsible for the review and approval of all human research using biobanks. They also review plans for the creation of biobanks. Some people feel that IRBs should have primary responsibility for oversight of biobanks.

Others argue that the biobank oversight extends beyond the traditional roles and responsibilities of IRBs. IRBs often focus more on the initial informed consent process and written consent forms than on other parts of research. They usually review studies from only one university. But biobank data is used for many studies. And data from biobanks are often shared among many institutions. In light of these concerns, some suggest that IRBs alone are not adequate. They believe we need new ways to oversee biobanks.

Some institutions have developed new systems of oversight, also called "governance," of biobanks. For example, one hospital biobank in the Midwest is governed by three external boards: the Ethics and Security Advisory Board, the Scientific Advisory Board, and the Community Advisory Group.

The regulation of biobanks differs around the world. Iceland and Estonia have enacted specific legislation to govern legal issues raised by biobanks, such as ownership of samples, informed consent, return of research results, benefit sharing and access to the biobank data.

6. Informed Consent

The informed consent process is the primary way IRBs make certain that people fully understand and agree to be a research participant.

Listed below are the components of informed consent:

- The person must have the ability to consent. A person who can't understand what is being proposed can't provide informed consent. For example, someone with severe Alzheimer's disease may be unable to provide informed consent.
- Consent must be given voluntarily, without pressure from the doctor or researcher.
- The person must have all the relevant information. The person must be told the purpose of the research and what will happen to him or her during the study. The consent must also discuss the risks and potential benefits of a study and whether it will cost anything to participate. The person must be told about how his or her information will be kept confidential.

With this information, a person can make a decision to participate or not based on his or her own needs, values, and interests. Asking permission in a careful and thoughtful way is the main way researchers and institutions like the University of California show respect for people.

Consent is More than Just a Form … It is a Process

Informed consent is not a one step process, although sometimes the written consent form becomes the exclusive focus of attention. The informed consent process is meant to ensure that those involved understand what they are agreeing to, including risks and benefits. It starts with the initial interaction between a researcher and a patient or potential research participant.

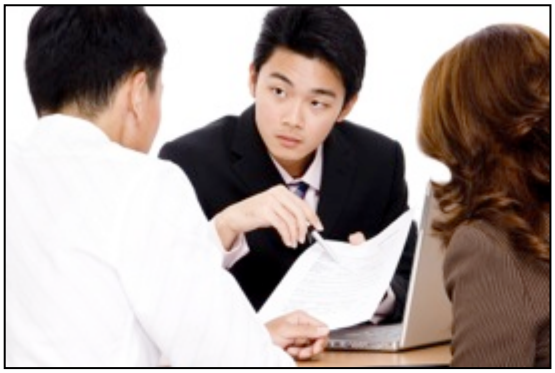

For biobanks, the consent process might involve ongoing communication with people who contribute to the biobank. Or it could involve communication in advance with the general public who might be involved.

Problems with Consent

Informed consent rules were designed for the following situation: a person is invited to participate in one research project. The project's risks and benefits can be fully described, because it is about to be carried out. Biobanks, on the other hand, can be used for many future research projects. Biobank researchers cannot know how a participant's sample or health information will be used in future research. A participant may be OK with sharing his health information today, but cannot guess what might happen in the future. This means

informed consent rules cannot fully protect potential participants.

Researchers, university leaders, and potential research participants are frustrated with the consent process. Consent forms are often long and complicated, and many patients find them confusing, overly time-consuming, or legalistic. Some patients may miss important details because they are overwhelmed by all the information. Other patients may feel pressure to sign the forms because they are used to following their doctor's instructions. Some people don't read the forms and make decisions based on whether they trust the hospital or doctor, or because a family member wants them to.

Is Consent Always Required?

Under current rules, informed consent is not required for researchers to use existing collections of anonymous tissue samples. A full informed consent process is generally required when the sample or the health information is not anonymous – that is, when the sample can be linked back to the name of the donor.

Can Consent Be Withdrawn?

Most biobanks are designed with no end date. However, current rules require that participants be allowed to withdraw consent to participate in research at any time. For biobanks, this might mean withdrawing the donor's sample from the library of available samples. Policies for withdrawal vary. The IRB might require that the researcher destroy the sample, make the sample completely anonymous so that the donor cannot be identified, or destroy information linked to the sample to prevent future use. But once biobank information has been shared with other researchers, it usually cannot be taken back.

7. How are Privacy and Confidentiality Protected?

Loss of privacy and breach of confidentiality are a major risk in biobanking. But we don't know how high the risk actually is. Often, people want to keep information about their health private. They want to have control over who has access to personal details. Privacy is important for people as individuals and also as members of a family or a community. Both California and the federal government have passed laws to protect the privacy of health information. Based on these laws, the University of California has produced policies and guidelines for its doctors and researchers. In biobanking, it is important to protect the confidentiality of both information from a sample and any health information linked to that sample.

Biobanks protect samples in several ways. Anonymized samples are permanently unlinked from the identity of the person who provided the blood or tissue. Or, samples may be deidentified. This means there is a code number that links the sample to the person's identity. Then only people who hold the key to the code know who the sample came from. (An example of deidentified samples is shown on the next page).

Studies often involve balancing privacy with potential research benefit. Difficulties can arise when rules that are intended to protect privacy conflict with research that requires personal information to meet its goals.

Protecting Medical Record Information by Removing Identifiable Information (Deidentification)

One way of protecting privacy is to remove all “identifiers” from patient information. Identifiers are pieces of information that reveal who a person is, such as names, birth dates, and addresses. Some researchers collect data using these identifiers, and later strip all identifying information from the collected data. This process is known as deidentification.

For example, data are often collected in the format shown in the table below. (The examples below do not use real names.)

Last name	First name	Sex	Date of birth	Date of diagnosis
Peterson	Andrew	Male	10/21/1945	11/30/1992
Chiu	Jane	Female	02/17/1930	01/25/1987
Gonzalez	Juana	Female	06/05/1937	07/27/1990
Smith	Harry	Male	05/22/1940	03/09/1997

The process of de-identification would result in the table below with real names and all exact dates removed:

Patient number	Sex	Age in 1990	Month disease was diagnosed
1	Male	45	11/1992
2	Female	60	01/1987
3	Female	53	07/1990
4	Male	50	03/1997

Is Genetics Information “Special?”

Some argue that genetic information is different than other kinds of information. Some view genetic information as special because genetic traits can be shared with your family members. Others view it as special because it can be used for identification (like at crime scenes or for paternity testing).

As discussed in this book, the use of genetic information can lead to negative outcomes for both individuals and groups. Privacy policies go a long way to safeguard against unintended uses. However, the possibility of a bad outcome remains. For example, if an employer finds out an employee is at risk of a costly, serious disease, that person may be passed over for promotion, in spite of laws prohibiting discrimination.

Info, Info Everywhere…

Privacy of health information is often hotly debated. Nevertheless, we give out our personal information every day to people we don’t even know. For example, when shopping online certain pieces of information are automatically collected, stored, and shared with others. This includes your name, contact information and even financial information. Every time you purchase your food at the grocery store with a consumer card, your personal information is stored in the grocery store’s database. The same is true when we use the Internet to search for information. Personal information may be collected by Google or Facebook. Why do we view health information as something that should be kept private when we give out so much personal information every day?

Is a Sample Ever Truly Private?

The more information researchers have about a given sample, the easier it is for them to identify the donor, even when the samples are anonymous. In reality, even a fully anonymous sample containing your DNA could be re-identified if you provided a sample of your DNA to match it against,

or if your DNA was in a criminal database or a genetic genealogy data base.

The U.S., Canada, and the United Kingdom all use DNA databanks for the purposes of criminal identification. The FBI's DNA database has been used as a tool to identify criminals and prove innocence. Military databases are used to help identify the remains of deceased military personnel.

Can a Person be Identified by DNA?

DNA is completely unique to each individual person. No one except you has your exact DNA makeup (unless you're an identical twin). Because DNA can be found in blood, semen, skin, bones and even sweat and saliva, it is a simple and effective way of identifying individuals. A sample the size of a pinhead is sufficient to collect enough DNA to identify someone. However, it is actually very difficult to find out the identity of a person who has participated in a biobank just from their DNA information. It is possible, but one would first need to have that person's DNA already to make a match.

8. What Privacy Rules Apply to Biobank Research?

Oversight of biobank research includes federal and state laws about privacy and use of medical records and health information. Privacy rules apply to clinical care in hospitals and clinics. Researchers who use samples and health information from hospitals and clinics also must observe the rules. Federal and California state privacy laws are then translated into policies at the University of California. The laws are a "minimum standard." University policies can go further. This section of the book reviews these laws and policies.

The Federal Privacy Rule: HIPAA and Medical Research

HIPAA stands for the Health Insurance Portability and Accountability Act. Since 2003, the federal HIPAA privacy rule has required that medical information about a person be kept confidential. It can't be shared with anyone who isn't authorized to get a patient's information. You may have noticed that you now receive information about your rights to privacy whenever you visit a doctor or health care provider.

HIPAA data security protections apply to the transfer of information from one doctor to another or from a hospital to an insurance company. HIPAA also requires that patients give written permission before their health information is shared with others, like a researcher. This is called giving "authorization" for use of health information.

For some studies, researchers may request a "HIPAA waiver" from the IRB or a special privacy committee. For example, if the study would otherwise be impossible to do, or if the researcher is not proposing to contact any patients but is only reviewing their medical records. In those cases, strict standards of confidentiality are used and the patient's real name is not kept. In these situations, the researcher may apply for a HIPAA waiver, which would allow them to conduct the research without contacting each person to obtain their written permission.

California Confidentiality of Medical Information Act (CMIA)

In California, the legislature passed a state law that controls to whom, when, and why medical information may be released. The Confidentiality of Medical Information Act works together with HIPAA to protect patients' right to privacy and to punish those who release information when it should have been kept private. Individual physicians or researchers as well as hospitals and clinics can be fined if medical information is inappropriately released.

At the University of California medical campuses, a fulltime Privacy Officer works with researchers and physicians to make sure that all involved are aware of and are following the federal and state rules. Additionally, if a patient's private health information is inappropriately released, the Privacy Officer is responsible for investigating what happened, reporting the details to the federal or state agency, and working with the people who made the mistake to make sure it does not happen again.

Rules Governing Sharing of Genetic Data with Federal Databases

In the U.S., data collected using funding from the government must be shared. The National Institutes of Health uses taxpayer money to support many studies, and it requires the researchers they support to share the information they collect. This includes genetic data. This allows many researchers, in both universities and private industry, to use the data that taxpayers have paid for. One federal database that collects genetic information is called dbGaP, which stands for "database of genotype (genes) and phenotype (disease)."

The rules governing sharing and use of data are strict:

- Only data from patients who have given written consent for their data to be shared are included in dbGaP.
- Researchers who want to use information stored in the dbGaP collection get special permission to access the information, and the researcher and his sponsoring institution must promise not to try to link dbGaP data with any individual.
- A special legal protection called a "Certificate of Confidentiality" covers all information in the dbGaP database. That certificate protects data from law suits. For example, if police thought a criminal's DNA was in the data base, the "Certificate of Confidentiality" would prevent them from getting access to the DNA information.

How the University of California Protects Health Information

UC keeps patients' information confidential in the following ways. Patient data can only be stored on certain computers and on computer systems that have a high level of security. All data are password protected. Laptop computers are protected with encryption technologies that make the information unreadable if a computer is lost or stolen.

All UC employees who take part in research are required to take special training about privacy laws, policies, and procedures. Training programs emphasize that everyone who works on a research study is responsible for protecting the privacy of the people in the study, and for keeping all data completely confidential. Patient records are monitored for improper use. Penalties for violating patient privacy are high. For example, employees who misuse a patient's personal health information can be fired.

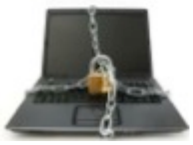

9. Should Results Discovered in Biobank Research be Offered to Participants?

Most University of California biobanks are created primarily for research. That means that the goal is to use samples to advance knowledge about health and disease. The purpose is not to provide treatment to individual people or patients. Treatments are provided as part of regular medical care (clinical care). In research, results learned in the course of doing a study are often not given back to participants because the information learned in a study is uncertain.

The Line between Research and Clinical Care

It used to be easier to draw a clear line between research and clinical care. However, two things are making that line less clear. First, biobanks are often created using leftover tissue samples from clinical care. Research projects collect samples from sick patients who are receiving treatment. Patients may confuse research with clinical care simply because the samples are collected in a hospital or clinic. This may happen even though doctors try to keep the line clear.

There is a second way in which the line between research and clinical care is blurring. New ways of doing research, such as genetic technologies, reveal more and more information about a research participant. Some of that information might be important for health. In the past, researchers studied one gene at a time. Now, technology makes it possible to study all of a person's genes at once. Whole genome sequencing is central to the field of personalized or precision medicine. But, when a person's whole genome is sequenced, it is possible that something health related will be revealed. Some people call these "incidental findings." For example, if researchers discover a gene that is related to a high risk of a fatal change in heart function, some people think the result should be offered to the biobank participant. That person could then take action. For example a cardiac device could be implanted.

Other people worry about the dangers of providing information learned during research. For one thing, research labs do not apply the same quality standards that clinical labs do. For that reason, federal rules require that only results of tests carried out in a certified lab may be given to people. Patients sometimes want to learn research results even though the results are preliminary and uncertain. But it often takes a long time to be certain of a research finding. For example, suppose that later research reveals that in some people the gene for heart function does not always create a risk of sudden death. In that case, returning results leads to unnecessary

surgery to implant the cardiac device and unnecessary worry.

The Shape of the Debate

There is a big debate about whether biobank participants should be notified about unexpected research results that might be important to them. This is called "Return of Results," and it is a very difficult issue.

Imagine the following example: Researchers are using biobank samples to search for causes of asthma. They do whole genome sequencing on a large number of samples. Several samples turn out to contain genetic changes that are known to increase a woman's risk for breast cancer. What should the researchers do? Should the information be offered to the biobank participant? This is a hard question. In some families with a known high risk of breast cancer, some people elect not to learn their genetic risk, even when it is offered to them. Other people want the information so they can take immediate action, such as the actress Angelina Jolie's recent decision to have her breasts surgically removed to prevent cancer.

Policy Options

Several ways of solving the dilemma have been proposed. Some ideas include changing the biobank informed consent process. Many IRBs will only allow results to be returned to participants if that person has given "up front" permission. But it is very difficult to ask people for permission about something that cannot be predicted ahead of time. Many people say "yes," they want to know "everything." But, when faced with learning they have a risk for an untreatable disease they change their minds. And how do researchers find out if the patient would not want to know information about an untreatable disease without letting him or her know that the information is now available?

Another idea is to provide biobank participants with "group" results. Many biobanks have newsletters that are mailed to participants or websites that provide updates. The fact that a new research result has been discovered could be shared with all research participants. Then people who were interested could call the biobank or could ask their doctor for advice.

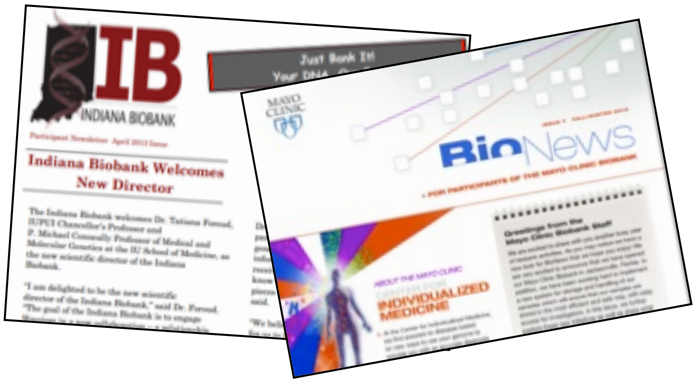

Who Should Give Back Results to Biobank Research Participants?

Another dilemma is who should give important research results back to patients. Researchers may not be medical doctors, and thus would not be able to explain what the result means for the patient's overall health, including if any tests or treatments were recommended.

And others worry that a primary care doctor may not be the right person to discuss the results with the patient. If a disease is very rare the primary care doctor may not know how to explain the condition and treatment options to a patient.

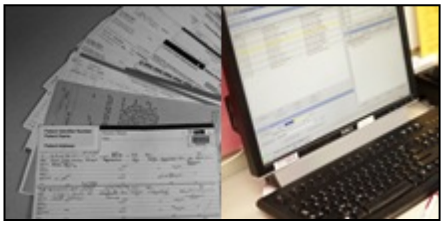

Risk of Discrimination

Some people are worried about discrimination if information from their genetic samples is shared, even with their physician. Research results may not become part of the "official" medical record. But doctor's visits and discussions with genetic counselors are part of the medical record. Once information about a possible condition is entered into the record, this information can be seen by anyone who has the patient's permission to search the medical record, such as life or disability insurance companies. If in the medical record, the information could result in patients being unable to get insurance. Of course the patient's permission to share the records is required. But patients may not fully understand the dangers of disclosure.

Finally, biobanks that do not keep links between a person's identity and their samples and health information are not able to return results. In those situations, the high value put on privacy protection makes it impossible to provide results to participants.

10. Biobanks and California Communities

In our deliberations, we need to consider the impact that biobanks will have on communities that have faced discrimination or racism in the history of the U.S. In the following pages, we discuss how different California communities may be affected by biobanking in more detail.

Race and Ethnicity

Biobanks often identify the race or ethnicity of a person who contributed a sample. This is helpful because different racial and ethnic groups have different kinds of health issues. At the same time, medical research that examines race can create the false impression that some races are biologically "superior" to others. This mistaken belief in eugenics can lead to real harm. In the past, people from minority groups were unjustly stigmatized or physically harmed because they were believed to be biologically "inferior."

In thinking about biobank research it is important to consider race and ethnicity. We must recognize how race and ethnicity can be useful for biobank research as well as how it can possibly lead to harm.

What is Race/Ethnicity?

Race and ethnicity are social groups that reflect ancestry. People from different parts of the world develop different languages, cultures, and customs. When they have children with others from the same ancestry group, this can lead to biological differences. This is why individuals from different racial or ethnic groups may look different in skin tone, height, or other characteristics.

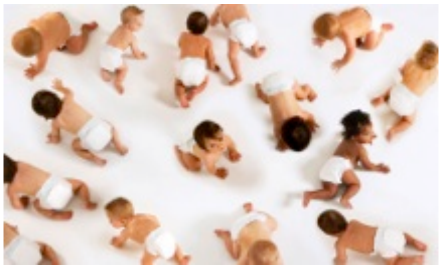

You may be surprised to hear that in spite of physical differences between different ethnic/racial groups, all racial/ethnic groups are very genetically and biologically similar to each other. Scientists have repeatedly shown that the biological differences of individuals within a racial group are much larger than biological differences between any two groups.

Cautions about Using Race/Ethnicity in Biobank Research

- May discourage participation in genetic research that is beneficial to everyone.
- Might lead to discrimination against certain groups.
- Could delay scientific progress by focusing on racial groups rather than on similarities and differences among individuals.
- May create too strong a focus on biology with less attention on how social conditions determine health status.

Race, Society, and Health

Even though people from all races and ethnicities are medically similar, ancestry makes a difference in how people live and are treated by others. People from different racial/ethnic groups may eat different foods or live in different neighborhoods. Different racial/ethnic groups may have differences in education, wealth, and power. Some groups may experience discrimination due to race and ethnicity. All of these factors – food, neighborhood, education, wealth, power, discrimination – are social factors that can influence health.

How Race/Ethnicity Can be Useful in Biobank Research

- To label divisions in the human population and make research more organized;
- To increase the involvement of under-represented minority populations in research;
- To inform studies about ancestry groups and migration patterns of the human population;
- To generate and explore race-specific connections between genetics, environment, disease, and lifestyle.
- To target populations for specific treatments or disease prevention.

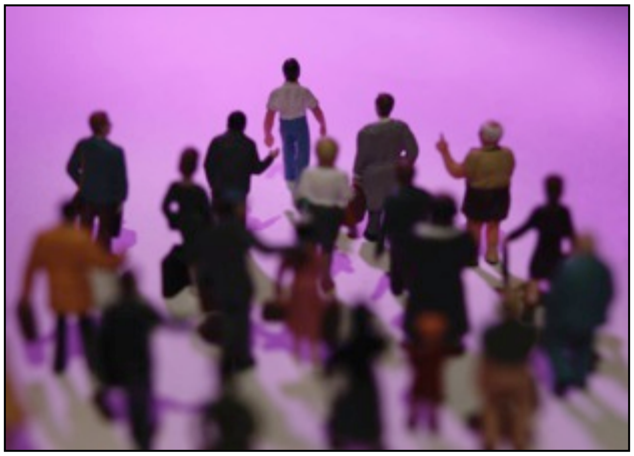

Controversy around the Use of Native Americans' DNA in Research

In 2004, the Havasupai Tribe brought a lawsuit against researchers at Arizona State University when they discovered that samples donated for diabetes research had been used for other purposes. Researchers were also using the tribe members' samples to look at human migration patterns and to look at genes for mental illness without their permission. The tribe sued Arizona State University because the researchers did not honor the promise they made about how samples would be used. In April 2010, a legal settlement awarded the Havasupai monetary compensation and the return of the DNA samples. More community discussion about questions of asking permission and justice for Native American and other vulnerable populations is needed. It is important for researchers to seek out the diverse views of participants and improve communication with and inclusion of vulnerable populations in genetic studies.

This incident suggests the importance of including the voices of Native Americans in designing biobanks. It also has broad implications for California, showing how groups can be harmed. It is critical to have the voices of those affected by the research included in the governance of biobanks.

Genes matter for health, too. Some genes occur more commonly in certain racial/ethnic groups, such as the gene related to sickle cell disease. There are also many questions about how social factors, such as wealth or power, lead to differences in health. Studying the health and biology of people from diverse racial/ethnic groups can be important for understanding how social and biological factors affect health.

When it comes to the health status of different racial/ethnic groups, scientists generally have shown that society matters more than the biology. But biology does play a role in certain diseases. It may be important to recognize race in some research projects. It is also important not to encourage racism. The box contains some considerations about the complex issue of race and biobank research.

The Challenge of Group Harms

In the U.S., racial and ethnic groups such as African Americans, Native Americans, and Latinos have suffered a legacy of racism and injustice. Those with disabilities are also subject to discrimination. These groups may be harmed by the use of their health information in research. For example, if research looks at the rate of mental illness in a certain California group, the entire group might be stigmatized. We call this a "group harm."

The system of human subjects protection described earlier focuses on protecting individual people from harm, not protecting groups, such as Native Americans or the disabled. The informed consent process helps people to protect themselves from potential harms. For example, a member of a certain Native American tribe might be asked to participate in a study of genetic links to alcoholism. If he or she objects to such research, they can say "no." However when a research project has implications for a group of people, or for an entire community, simply asking individuals for consent may not be enough to make certain the community is not harmed. Even if half the people in a community say "no" to participation, the research that makes comparisons by race continues. The

reputation of the entire community can be affected.

In some cases, IRBs consider group harms. Community guidance about group harms is needed. In addition to protecting individuals from the risks of research, we must consider the ways that different groups in our society could be harmed or benefit from research. Community engagement gives everyone the opportunity to discuss concerns about biobanking research.

Prenatal Testing

Genetic research using biobanks may add to the number of genetic traits we are able to detect early in pregnancy. People with disabilities and disability advocates worry that as genetic technologies advance, more and more genetic traits will be labeled as "abnormal." Advocates are concerned that prenatal genetic testing might lead to more decisions to terminate pregnancies. If the number of people with a condition decreases, will the services available to help those with the disability also decrease?

11. Commercialization, Benefit Sharing, and Ownership

One goal of medical research is to provide new treatments and medicines. Private companies frequently have a role in this process. Companies invest in research that brings new treatments and medicines to market. The new medicines are then sold for a profit. The development of new treatments and medicines is a very costly process. It is also a risky process because many promising new treatments and medicines do not make it to the market. Drugs based on discoveries may not be effective or they may be unsafe. The same uncertainty surrounds other medical products, such as new diagnostic tests.

The potential for profit encourages companies to take risks. But it also raises questions about who owns biobank samples, who should be able to access biobank samples and data, and who should benefit from the results of research?

Who Benefits from Biobank Research?

Should individual biobank participants benefit from commercialization of research using biobank samples? This question has generated controversy, as in the case of Henrietta Lacks reported above. It is <u>very</u> rare that a single person's blood or tissue leads to a commercially profitable product. However, cases like this raise the question of who "owns" biobank samples. Are our bodies and donated tissue our "property"? The courts consistently have found that people do not own their samples and do not have any rights to profits from these samples. However, there is still the issue of who should benefit from the knowledge and products that such donations help create? Are there other ways to provide benefits back to donors or their communities, such as by contributing to community health programs? These debates are ongoing.

Some people argue that thinking about human tissue in terms of ownership or financial return is the wrong approach. They propose a model based on the concept of "stewardship." In stewardship models research participants or patients provide their samples to an organization like UC without expectation of personal benefit. The biobank also does not "own" the samples, but has the responsibility to hold the

samples "in trust" to benefit the health of the broader community. In this model samples cannot be bought and sold. The Genetic Alliance Biobank is owned and managed by patient advocates who represent people with genetic diseases. Researchers apply to a disease advocacy organization for permission to use biobank samples.

12. What Role Have Communities Played in Biobank Oversight?

This section of the book talks about how to balance the wishes of each biobank participant with judgments made by the larger community that shares the risks and benefits of biobank research.

Earlier, we described the many laws and regulations that work together to protect participants in biobank research. The U.S. Human Subjects Protection system protects individual research participants from harm. Generally, the system tries to make sure each person is able to make wise choices about participation in research. This allows each person to decide according to his or her personal values. Informed consent is an important part of this process. That's why people are given detailed brochures and informed consent documents to read and sign.

But biobanking presents a challenge to our current system of human subjects protection for several reasons. The current system was designed for specific research projects. Biobanks are different; they are "resources" to help future research. No one knows exactly how the biobank will be used later. Issues and problems may come up years and years after a person originally enters the biobank. For example new research findings create the dilemma of when and how to return unexpected findings to biobank participants.

Many biobanks were established before techniques like genetic sequencing were developed. Thus relying on a person's initial wishes may be difficult. Research participants may have said "no" to getting any information back from a study. But doctors now believe that a particular piece of information could be lifesaving. This creates a dilemma for researchers and UC. Some people might argue that the new technologies should not be used to study existing samples if specific permission was not given.

Another example is data sharing. Many years ago it was uncommon to share data among many researchers from different universities. Now data sharing is required by the U.S. National Institutes of Health. Going back to each person and asking him or her to weigh in on each new issue is costly. And it may not be possible to locate people; some will have died. Should data be shared or should patients be contacted and asked for their permission?

Some biobanks have created "Community Advisory Boards" to help address these issues. These boards often include members of the local community, such as patients, religious leaders, business people, etc. When a board is in place, researchers and hospital or clinic leaders can take challenging ethical questions to the board and ask for advice.

One example of a biobank that has an advisory board is the Mayo Clinic Biobank (Minnesota). At the Mayo Clinic, some members of the board originally participated in a deliberative community engagement like the one you are participating in now. The Mayo Clinic Biobank board has helped

researchers and Mayo Clinic doctors answer questions about whether a specific research finding should be offered to biobank participants. One study that used the biobank revealed information about a small number of biobank participants. It showed that these people had a gene variant linked to blood clots. This means that the patients might need different drug treatments following surgery or might not want to take certain drugs that also raise the risk of blood clots, such as birth control pills. The community board had several meetings with biobank leadership and Mayo Clinic experts in blood clots. The community board suggested a limited number of situations in which biobank participants should be notified of their risk and recommended specific ways to contact participants, commenting on letters and procedures. Mayo Clinic leaders considered the community's views and worked closely with the board as final policies were developed.

In this example, the board provided the perspective of non-experts who understood the concerns of community members. The board members became the voice of the larger community. This allows the community a direct and formal role in biobank policy. Having a community board in place changes the way we honor the patient's individual choice and control. In a biobank with an active community board, research participants consent to being governed by others.

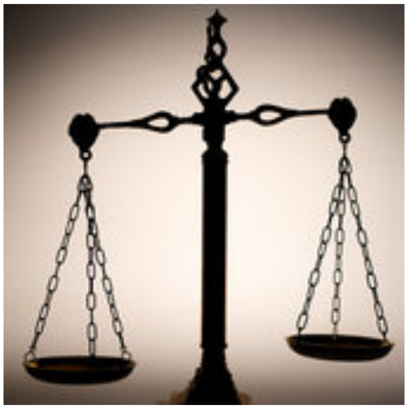

13. Future Governance of University of California Biobanks

There are a number of decisions about the future management of biobanks that University of California leaders will need to make. Five University of California campuses with hospitals and medical schools (Davis, Irvine, Los Angeles, San Diego, San Francisco) have come together to form a group called UC BRAID that is helping guide biobanking decisions. UC BRAID includes scientists, administrators, physicians, lawyers, and leaders of the university's IRBs. One goal of UC BRAID is to make it easier and less expensive for University of California researchers to use and share biobank data and samples. Another goal of UC Braid is to make sure the interests of California biobank participants are fully protected. UC BRAID does not currently include community members, but seeks community input.

How should the community be involved? Biobank research is overseen by University of California IRBs. Each campus has its own IRB, and they also work together on policy. However, few members of the California community participate in IRBs. The federal rules require only one community member on each IRB. Since biobank research can be very complicated, additional community guidance may be needed. In particular, the perspectives of those groups who are most vulnerable to harm must be included.

Balancing the benefits of research with the necessary protections and safeguards for biobanks is difficult. In the past, it was

uncommon to ask the community for advice about research biobanks.

The EngageUC project seeks to change that. This community event is to work with you to get your thoughts about how to govern the University of California biobanks.

During the Community Engagement We Will Talk about the Following Questions:

- What kinds of biobanks are acceptable to California residents?
- Overall, what is the right balance between protection of biobank participants and potential benefits from research?
- What thoughts and concerns do California residents have about the security, privacy and confidentiality of medical information and samples used in research?
- One goal of a biobank is data sharing to make research possible. What thoughts and concerns do community members have about which researchers have access to biobank data? What about industry or government scientists? Those in other countries?
- When and how should University of California patients be asked permission for use of their samples, including leftover tissues, and health information? EngageUC will be doing a study comparing different ways of explaining biobanks and inviting people to participate. Our discussions during the Community Engagement events will help plan that research study.
- Research rules change over time. Some existing samples were collected in the past under different rules than exist today. Some of these collections are very old or from patients with very deadly diseases, and thus the people who provided the samples may no longer be living. What thoughts and concerns do community members have about these collections? How should such collections be managed?
- What about priorities for biobank research? Should community members have a voice in deciding which types of research are most important or timely? If so, how?
- Should the University of California develop a community advisory board (or similar process) that meets regularly to advise UC, including its scientists and doctors, about biobank research? If so, what should the process be? How should community members be selected? How can the California community be involved in biobank governance in a meaningful way?

Next Steps

These questions are only a starting point. We look forward to hearing your questions and listening to your suggestions.

Your recommendations will be given to UC BRAID and leaders in the University of California Office of the President. They will also be shared and discussed with community organizations in California.

Thank you for reviewing this booklet!

14. Glossary

Anonymous/anonymized samples and/or data: The link between samples and data collected by a biobank and the identity of the donor never existed (following donation) or has been destroyed so that it would be impossible under any circumstances to re-link the sample and data with the donor. If a link is kept the sample is not truly anonymized.

Benefit sharing: Sharing the rewards derived from research into human genetics with tissue donors, patients and/or humankind.

Biobank: A collection of biological samples connected to health information.

Biological samples (or Biospecimen): Materials from the human body, such as tissue, blood, plasma, and urine that can be used for diagnosis and analysis of patients.

Clinical Research: Study of a drug, biological compound, or device in human subjects to determine its effect on health or disease.

Clinical Trial: Researchers test hypotheses with human participants about the effect of a particular intervention (such as a new drug) upon a disease condition in comparison with another drug or an inactive substance (placebo) upon a control group that lacks the disease condition.

Commercialization: To arrange activities and products for the purpose of gaining profit and/or stimulating investment.

Confidentiality: The protection of information from being circulated outside of an authorized group.

Data: Observations and values measured and recorded as a result of a scientific investigation. In biobanking, data is the collection of information available from biological samples as well as any additional patient information related to the samples. The term “big data” is used to describe the wave of genetic technologies that require tens of thousands of samples in order to study how very small genetic differences among individuals might lead to different illnesses.

Deidentification: Biological samples are often linked to health information from the medical record. The health information makes the samples more valuable to researchers. Some of this health information, such as a person’s name and the date they were diagnosed, might identify the person. Deidentification is the process by which researchers strip all identifying information from the samples they collect, in order to protect an individual’s privacy.

Deliberation: Respectful discussion in which participants offer views, reasons and other representations of their views in an attempt to understand each other and determine agreements and disagreements.

Deliberative democracy: A form of representative democracy which involves groups of citizens who discuss and decide policy issues; an approach focused on enhancing the nature and form of political participation.

DNA: An abbreviation for deoxyribonucleic acid, the essential molecule of heredity. The twisted ladder of the base pairs (also more famously known as the double helix) of the DNA molecule contains the chemically coded instructions to construct and maintain a living organism.

Drug development: The process of bringing a drug discovery through the stages needed for it to be tested in a human clinical trial.

Eugenics: Term coined in 1883 by Sir Francis Galton (1822-1911) meaning "wellborn." The term is rooted in the belief that nature is more important than nurture and the social philosophy that humanity can be improved through intervention. Historically associated with Nazi abuses such as the extermination of certain populations, but today is debated with reference to reproductive and genetic technologies.

Gene: Genes are both units of inheritance and encoded messages for the creation of a functional unit in a cell. These functional units influence, to varying degrees, an organism's appearance, its metabolism and sometimes even its behavior, among other things.

Genetic disease: A disease caused (or strongly influenced) by abnormalities in an individual's genetic material (genome). For example, Huntington's disease, Tay-Sachs disease and Parkinson's disease are all considered genetic diseases or disorders.

Genetic markers: A specific genetic trait that has a molecular feature that can be used to measure or indicate the effects, progress or potential to develop a disease.

Genome: (1) The full set of genes carried by a single organism and (2) the set carried by that organism's species. The precise ordering of nucleotides (one molecular component of DNA) in organisms' genomes is the foundation of life's diversity. It dictates whether an organism is human or another species, such as yeast, rice, wombat, gnat or fruit fly, all of which have their own genomes and either are or could be the focus of a genome project. Because all organisms are related through similarities in DNA sequences, insights gained from nonhuman genomes often lead to new knowledge about human biology.

Genetic testing: Any procedure to determine whether a person has a gene that is associated with a disease or characteristic.

Genomics: A discipline dedicated to the understanding of the entire genetic information content of an organism and its relation to environment and the whole organism.

Governance: The use of power to direct behavior through law, policy, professional practice standards or social conventions.

HIPAA: The Health Insurance Portability and Accountability Act of 2003. This federal privacy rule requires that medical information about a person be kept confidential. In other words, it cannot be shared with anyone who isn't authorized to receive it. For example, one doctor may want to share information about your medical care with another doctor involved in your medical care or with a research study. Because of HIPAA, the doctor is required to ask for your written permission to do so.

Institutional Review Board (IRB): Committees at health care institutions, universities, and some corporate environments that are responsible for protecting human subjects involved in research through multidisciplinary review of research protocols. Where the IRB is not satisfied that the protocol meets established ethical standards, it can prevent the research from starting or, where concerns arise in relation to ongoing research, from continuing.

Medical record: The information entered and maintained by your doctors and other health care professionals, which documents your health history, medical visits, drugs you have been administered, test results, and other details of your medical care, such as x-rays or surgical reports. Doctors are required to enter and store complete, accurate records of their patients' care. However, these records often include sensitive, personal information and are considered the property of the patient who can obtain a copy at any time.

Mutation: Errors in copying base pairs of DNA when cells are dividing. Most mutations are silent, meaning that they do not change the protein specified by the gene. Other mutations are fatal to embryos or the basis for the evolution of new characteristics.

Patient: A person receiving or registered to receive health care.

Personalized (or precision) medicine: The use of detailed information about a patient's genotype or level of gene expression and a patient's clinical data in order to select a medication, therapy or preventative measure that is particularly suited to that patient.

Personal health information (PHI): The information collected by a doctor or other health care professional to identify an individual and decide how to provide them with medical care. Generally, personal health information, or PHI, includes demographic information, medical history, test results, and even insurance information.

Pharmacogenomics (or pharmacogenetics): The study of the genetic basis for responses to drug therapies, with the intention to ensure maximum efficacy with minimal adverse effects. It is the basis of claims about personalized medicine, in which drugs and drug combinations are optimized for each individual's unique genetic makeup.

Population health: The measures of health, including lack of disease, longevity and other factors, across the members of a group.

Stigmatization: To characterize or brand as disgraceful or undesirable.

Tissue: Human cells from any part of the body. This includes blood, urine, skin, and saliva.

Whole genome sequencing: The process by which scientists are able to create a full copy of every piece of an individual's DNA.

15. Acknowledgements

The following people helped create this Briefing Book and the Community Engagement:

UCSF

Elizabeth Boyd
Daniel Dohan
Megan Dowdell
Jen Hult
Barbara Koenig
Jessaca Machado

UCLA

Arleen Brown
Blanca Corea
Sarah Dry
Arturo Martinez
Stefanie Vassar

With thanks to:

Michael Burgess
University of British Columbia, Canada

Holly Longstaff
University of British Columbia, Canada

Kieran O'Doherty
University of Guelph, Canada

And to: BITTS - Berkeley Interpreting, Translation & Transcription Services for translating the booklet into Spanish

This project was supported by the National Center for Advancing Translational Sciences, National Institutes of Health, through UCSF-CTSI Grant Number UL1 TR000004-07S2, with additional support through UCLA-CTSI Grant Number UL1 TR000124. Its contents are solely the responsibility of the authors and do not necessarily represent the official views of the NIH.

Fair copying of this publication is permitted for teaching, review or research.
