# Supplementary material for: Community recommendations on biobank governance: Results from a deliberative community engagement in California
Source: PLoS One. 2017 Feb 24;12(2):e0172582. doi: 10.1371/journal.pone.0172582 (PMC5325297; doi:10.1371/journal.pone.0172582)
Supplement: S1 Table — (PDF) [file pone.0172582.s002.pdf]

S1 Table. Agenda for Los Angeles Deliberative Community Event

First weekend - Saturday June 15th, 2013

Education and information

Time allotted	Task
9:00-9:30	Check in/Review of Information Sheet/Survey
9:30 -9:45	Welcome!! (Judith Gasson)
9:45-10:40	Introductions
10:40-11:00	Overview of event and ground rules
11:00-11:15	*Break*
Expert presentations. Please note that all 15-20 minute talks will be followed by 10-15 minutes of Q&A (Speaker information is in packets)	
11:15-11:45	Talk 1: What is biobanking and how does it work? (Sarah Dry)
11:45-12:15	Talk 2: What are the benefits of biobank (and related) research? Part 1 (Michele Rakoff)
12:15-12:45	Talk 3: What are the benefits of biobank (and related) research? Part 2 (Satiro De Oliveira)
12:45-1:30	*Lunch*
1:30-2:00	Talk 4: What protections are in place for data and samples? (Rachel Nosowsky)
2:00-2:30	Talk 5: What concerns do people have about sharing their biological materials and data with biobanks, and about research done later by those who use biobanks? (Nanibaa' Garrison)
2:30-3:00	Talk 6: What models exist for community governance of biobanks? (Julie Harris-Wai)
3:00-3:20	*Break*
3:20-4:20	Speaker panel and Q & A period
4:20-5:00	Large group discussion of expert presentations and overview of tasks and goals for Sunday
5:00	Checkout

Sunday June 16th, 2013

Exploring issues and developing recommendations

Time allotted	Task
10:00-10:15	Overview of tasks and goals for the day
10:15-10:45	Large group stakeholder discussion
10:45-11:00	*Move into small groups and short break*
11:00-12:00	Identifying general hopes and concerns for biobanks in small groups
12:00-12:45	Report back to large group (5 min presentations and 10 min discussion for each group)
12:45-1:30	*Lunch*
1:30-2:30	Biobank models –small group discussion
2:30-3:00	Recommendations for biobank models in large group
3:00-3:15	*Break*
3:15-4:15	Return of research results - small group discussion
4:15-4:45	Recommendations for return of research results in large group
4:45-5:00	Checkout, evaluation, and explanation of handouts to review before second weekend

Second weekend - Saturday June 29th, 2013

Developing Recommendations

Time allotted	Task
9:00-9:15	Welcome back and overview of second weekend tasks and goals
9:30 -10:45	Ideal models of informed consent process–small group discussion
10:45-11:00	*Break*
11:00-12:00	Recommendations for ideal models of informed consent process in large group
12:00-12:45	*Lunch*
12:45-1:45	Biobank sharing (samples & data) -small group discussion
1:45-1:55	*Break*
1:55-2:50	Recommendations for biobank sharing (samples & data) in large group
2:50-3:00	*Break*
3:00-4:00	Features of trustworthy biobank oversight–small group discussion
4:00-4:45	Recommendations for trustworthy oversight in large group
4:45-5:00	Checkout

Sunday June 30th, 2013

Developing and Voting on Recommendations

Time allotted	Task
10:00-10:15	Overview of tasks and goals for Sunday<br>Review of next steps for EngageUC biobanking project.
10:15-11:15	The role of community in biobank governance –small group discussion
11:15-11:30	*Break*
11:30-12:00	Recommendations for the role of community in biobank governance in large group
12:00-12:45	*Lunch* All sessions after lunch are in the large group
12:45-1:15	Voting on recommendations for the role of community in biobank governance
1:15-1:45	Voting on recommendations for trustworthy oversight
1:45-2:15	Voting on recommendations for biobank models
2:15-2:45	Voting on recommendations for sample and data sharing
2:45-3:00	*Break*
3:00-3:30	Voting on recommendations for return of research results
3:30-4:00	Voting on recommendations for ideal models of informed consent process
4:00-4:30	Discussion of final recommendations with panel of UC leaders.<br>(Dan Mercola, UCI and Michael Caligiuri, UCSD).
4:30-5:00	Wrap up –Evaluation, biobanking survey, goodbye, and thank you!
