# Supplementary material for: Community recommendations on biobank governance: Results from a deliberative community engagement in California
Source: PLoS One. 2017 Feb 24;12(2):e0172582. doi: 10.1371/journal.pone.0172582 (PMC5325297; doi:10.1371/journal.pone.0172582)
Supplement: S2 Table — (PDF) [file pone.0172582.s003.pdf]

S2 Table. Agenda for San Francisco Deliberative Community Event

First weekend - Saturday, September 21st, 2013

Education and information

Time allotted	Task
9:00-9:30	Check in/Review of Information Sheet/Survey
9:30 -9:45	Welcome!! (Kevin Grumbach)
9:45-10:40	Introductions
10:40-11:00	Overview of event and ground rules
11:00-11:15	Break
11:15-11:20<br>11:20-11:30	Description of the UC System (Elizabeth Boyd)<br>Description of the EngageUC project (Dan Dohan)
Expert presentations. Please note that all 10-15 minute talks will be followed by 5-10 minutes of Q&A (Speaker information is in packets)	
11:30-11:55	Talk 1: **What is biobanking and how does it work?**<br>(Sarah Dry)
11:55-12:15	Talk 2: **What are the benefits of biobank research?**<br>Patient perspective (Diane Borrison)
12:15-12:40	Talk 3: **What are the benefits of biobank research?**<br>Researcher perspective (Margaret Tempero)
12:40 – 1:05	Speaker panel and Q & A period – Talks #1-3
1:05 – 1:50	Lunch
1:50 – 2:15	Talk 4: **What protections are in place for data and samples?** (Rachel Nosowsky and Eric Mah)
2:15 – 2:40	Talk 5: **What concerns do people have about biobanks?**<br>(Sandra Lee)
2:40 – 3:00	Talk 6: **What models exist for community governance of biobanks?** (David Winickoff)
3:00 – 3:15	Break

3:15 – 3:45	Speaker panel and Q & A period - Talks #4-6
3:45 – 4:00<br>4:00 – 4:30<br>4:30 – 4:50	Large group discussion of expert presentations<br>***Stakeholders*** – large group discussion<br>Overview of tasks and goals for Sunday
4:50 - 5:00	Checkout

Sunday, September 22nd, 2013

Exploring issues and developing recommendations

Time allotted	Task
10:00 - 10:15	Overview of tasks and goals for the day
10:15 - 10:25	Move into small groups
10:25 - 11:10	***Hopes and Concerns for Biobanks*** - small group discussion
11:10 - 11:15	Move back to large group
11:15-12:00	Report back ***Hopes and Concerns*** to large group (5 min presentations and 10 min discussion for each group)
12:00-12:45<br>12:45-12:50	Lunch<br>Move into small groups
12:50 – 1:50	***Biobank models*** –small group discussion
1:50 - 1:55	Move back to large group
1:55 – 2:55	Recommendations for ***Biobank Models*** **-** large group
2:55 – 3:10	Break and move into small groups
3:10 - 4:10	***Sample and Data Sharing*** - small group discussion
4:10 – 4:15	Move back to large group
4:15 – 4:45	Recommendations for ***Sample and Data Sharing*** - large group
4:45 - 5:00	Checkout, evaluation, and explanation of handouts to review before second weekend

Second weekend - Saturday, October 5, 2013

Developing Recommendations

Time allotted	Task
9:00-9:15	Welcome back and overview of second weekend tasks and goals
9:30 -10:45	Ideal models of informed consent process–small group discussion
10:45-11:00	*Break*
11:00-12:00	Recommendations for ideal models of informed consent process in large group
12:00-12:45	*Lunch*
12:45-1:45	Biobank sharing (samples & data) -small group discussion
1:45-1:55	*Break*
1:55-2:50	Recommendations for biobank sharing (samples & data) in large group
2:50-3:00	*Break*
3:00-4:00	Features of trustworthy biobank oversight–small group discussion
4:00-4:45	Recommendations for trustworthy oversight in large group
4:45-5:00	Checkout

Sunday, October 6, 2013

Developing and Voting on Recommendations

Time allotted	Task
10:00-10:15	Overview of tasks and goals for Sunday<br>Review of next steps for EngageUC biobanking project.
10:15-11:15	The role of community in biobank governance –small group discussion
11:15-11:30	*Break*
11:30-12:00	Recommendations for the role of community in biobank governance in large group
12:00-12:45	*Lunch* All sessions after lunch are in the large group
12:45-1:15	Voting on recommendations for the role of community in biobank governance
1:15-1:45	Voting on recommendations for trustworthy oversight
1:45-2:15	Voting on recommendations for biobank models
2:15-2:45	Voting on recommendations for sample and data sharing
2:45-3:00	*Break*
3:00-3:30	Voting on recommendations for return of research results
3:30-4:00	Voting on recommendations for ideal models of informed consent process
4:00-4:30	Discussion of final recommendations with panel of UC leaders.<br>(Arleen Brown, UCLA, Elizabeth Boyd, UCR, Claire Brindis, UCSF, Regina Gandour-Edwards, UCD)
4:30-5:00	Wrap up –Evaluation, biobanking survey, goodbye, and thank you!
