# Supplementary material for: Community recommendations on biobank governance: Results from a deliberative community engagement in California
Source: PLoS One. 2017 Feb 24;12(2):e0172582. doi: 10.1371/journal.pone.0172582 (PMC5325297; doi:10.1371/journal.pone.0172582)
Supplement: S3 Table — (PDF) [file pone.0172582.s004.pdf]

S3 Table. Deliberant-Generated Recommendations that were Highly Supported Across DCE Sites (Included in Analyses)

Los Angeles Recommendations and Votes (Yes/No/Abstain)

**Community governance**		
LA1	There should be meaningful and transparent involvement of communities in the governance and oversight of biobanks.	26/0/0
LA2	Communities should be represented by a diverse, trustworthy, informed group of the general public.	25/1/0
LA3	There should be multiple forms of ongoing public education and communication about biobanks and research progress (e.g., newsletters, websites, TV)	25/1/0
**Trustworthy oversight**		
LA4	Oversight should be conducted by multiple, unbiased stakeholders (e.g., community members, scientists, medical professionals, lawyers)	22/2/2
LA5	Misuse should be identified through monitoring, and there should be consequences for misuse of samples and patient information.	26/0/0
**Sample and data sharing**		
LA6	UC should support sharing samples and data among researchers for the public good despite the risks, provided there is good ethical governance.	25/0/1
LA7	Research results should be shared back to research programs and clinical organizations	23/2/1
**Informed consent**		
LA8	Consent forms should be clear and use simple language, and permissions should be obtained as early as possible in the process	25/0/1
LA9	Consent should be an interactive process with an informed person, when the donor is not stressed.	24/1/1
**Return of research results**		
LA10	Participants should have a choice upfront about whether to receive results	23/2/1

San Francisco Recommendations and Votes (Yes/No/Abstain)

**Community governance**		
SF1	The community's role should include increased involvement with the IRB and a community board that provides recommendations to the governing board of the biobank.	22-0-3
SF2	The community must have a meaningful role in the oversight of UC biobanks.	24-0-1
SF3	The public should be made aware of biobanking and research through education, e.g. videos, events, etc.	25-0-0
SF4	Community representatives should be committed; they should represent California's diversity; membership should rotate; they should be independent of UC and other stakeholders, i.e. pharmaceutical companies	23-1-1
**Trustworthy oversight**		
SF5	Trustworthy oversight must include the IRB, the biobank, the community, and UC.	21-2-2
SF6	There must be monitoring and consequences for improper handling or use of data or samples, e.g. abuse, mishandling, release of information	23-1-1
**Sample and data sharing**		
SF7	UC should share **data** among all researchers as long as it advances research and there is good oversight.	22-2-1
SF8	UC can share **samples** among all researchers as long as it advances research and there is good oversight.	22-1-2
SF9	If data and samples are shared outside of UC, any information or findings should be shared back to UC.	24-0-1
**Informed consent**		
SF10	Consent forms must be written clearly, simply, in large fonts, and in the donor's preferred language.	24-0-1
SF11	Consent must be obtained by a trusted, knowledgeable person who has time to answer questions.	24-1-0
SF12	Consent for donation must be collected at a time when people are less stressed, worried, or pre-occupied.	24-0-1
**Return of research results**		
SF13	Receiving individual research results is a personal choice; donors should be allowed to opt-in or opt-out of receiving results.	24-0-1
